# Evolution of DS-1-like G1P[8] double-gene reassortant rotavirus A strains causing gastroenteritis in children in Vietnam in 2012/2013

**DOI:** 10.1007/s00705-016-3155-6

**Published:** 2016-11-23

**Authors:** Toyoko Nakagomi, Minh Quang Nguyen, Punita Gauchan, Chantal Ama Agbemabiese, Miho Kaneko, Loan Phuong Do, Thiem Dinh Vu, Osamu Nakagomi

**Affiliations:** 1 Department of Molecular Epidemiology, Graduate School of Biomedical Sciences, Nagasaki University, Nagasaki, 852-8523 Japan; 2 Department of Epidemiology, National Institute of Hygiene and Epidemiology, Hanoi, Vietnam; 3 Department of Virology, National Institute of Hygiene and Epidemiology, Hanoi, Vietnam

## Abstract

Rotavirus A (RVA) strains, a leading cause of severe gastroenteritis in children worldwide, commonly possess the Wa or DS-1 genotype constellations. During a hospital-based study conducted in Hanoi, Vietnam, in the 2012-2013 rotavirus season, G1P[8] strains with a virtually identical short RNA migration pattern were detected in 20 (14%) of 141 rotavirus-positive samples. Two representatives of these strains were shown by whole-genome sequencing to be double-gene reassortants possessing the genotype constellation of G1-P[8]-I2-R2-C2-M2-A2-N2-T2-E2-H2. Sequencing and a database search revealed that these Vietnamese G1P[8] double-gene reassortant strains shared an immediate ancestor with a locally circulating G2P[4] strain in all of the inner-capsid and non-structural protein genes, whereas they were more closely related in the VP7 and VP4 genes to a Chinese G1P[8] strain and a Chinese G3P[8] strain, respectively, than to locally circulating G1P[8] strains. Despite the marked similarity between Japanese and Thai G1P[8] double-gene reassortant strains, phylogenetic analysis suggested that the Vietnamese and Japanese/Thai G1P[8] double-gene reassortant strains originated from independent reassortment events. Clinically, children infected with Vietnamese G1P[8] double-gene reassortant strains experienced severe diarrhoea, but it was not more severe than that in children infected with ordinary G1P[8] strains. In conclusion, Vietnamese G1P[8] double-gene reassortant strains originated from a locally circulating G2P[4] strain and caused severe diarrhoea, but there was no evidence of increased virulence.

**Electronic supplementary material:**

The online version of this article (doi:10.1007/s00705-016-3155-6) contains supplementary material, which is available to authorized users.

## Introduction

Rotavirus A (RVA) is a leading cause of severe gastroenteritis in children worldwide [1]. RVA, a member of the genus *Rotavirus* within the family *Reoviridae*, has a genome comprising 11 segments of double-stranded RNA that encode six structural viral proteins (VP1-VP4, VP6, and VP7) and six non-structural proteins (NSP1-NSP5/6) [2]. Each of these genes is differentiated into genotypes according to a predefined nucleotide sequence identity cutoff value [3–5]. This classification system denotes the VP7-VP4-VP6-VP1-VP2-VP3-NSP1-NSP2-NSP3-NSP4-NSP5/6 genes as the descriptor Gx-P[x]-Ix-Rx-Cx-Mx-Ax-Nx-Tx-Ex-Hx (where x represents a genotype number) [3–5]. Most human RVA strains can be classified into three genotype constellations, Wa, DS-1 and AU-1, which are described as G1/3/4/9-P[8]-I1-R1-C1-M1-A1-N1-T1-E1-H1, G2-P[4]-I2-R2-C2-M2-A2-N2-T2-E2-H2, and G3-P[9]-I3-R3-C3-M3-A3-N3-T3-E3-H3, respectively [3, 6].

In 2012, novel double-gene reassortant strains with the genotype constellation G1-P[8]-I2-R2-C2-M2-A2-N2-T2-E2-H2 emerged in Japan [7–9]. Upon whole-genome analysis of these double-gene reassortant strains, Fujii et al. [9] concluded that the strains were of clonal origin and spread throughout the entire country with a detection rate of 31-63%. Similarly, in Thailand, three G1P[8] double-gene reassortant strains were sporadically detected in 2013 [10]. All 11 genes of the Thai strains were reported to be closely related to each other and to those of Japanese G1P[8] double-gene reassortant strains, and they were considered to have originated from a recent common ancestor [10].

In the 2012-2013 rotavirus season in Hanoi, Vietnam, where we conducted a rotavirus study, over a quarter of G1P[8] rotavirus-positive samples exhibited short RNA migration patterns upon polyacrylamide gel electrophoresis. As short RNA migration patterns are usually associated with the DS-1-like genotype constellation [11], we determined the whole genotype constellation of representative Vietnamese G1P[8] strains to understand how they emerged and how they were related to the Japanese and Thai G1P[8] double-gene reassortant strains. In addition, it was explored whether the G1P[8] double-gene reassortant strains caused more-severe disease in children than ordinary G1P[8] strains

## Materials and methods

### Study specimens and case patients

A cross-sectional study was performed at Saint Paul Hospital and Bach Mai Hospital, Hanoi, Vietnam, from November 2012 to June 2013 (the 2012/2013 rotavirus season in Hanoi). Briefly, faecal specimens were collected from all children less than two years of age who were hospitalised for acute diarrhoea, which was defined as three or more looser-than-normal stool passages or watery diarrhoea during the preceding 24 hours. For each faecal specimen, a 10% suspension (w/v) was made in phosphate-buffered saline (pH 7.2), and tested for rotavirus antigen using an enzyme-linked immunosorbent assay (Premier Rotaclone, Meridian Bioscience, Inc., OH, USA) according to the manufacturer’s instructions.

Demographic data for the enrolled patients were collected together with information about signs and symptoms they presented at the time of hospitalisation in order to calculate the severity score of diarrhoea according to Vesikari’s 20 point scale [12]. Informed consent was obtained from the parents or guardians of the enrolled patients, and the study was approved by the institutional review boards of the participating hospitals as well as the National Institute of Hygiene and Epidemiology, Vietnam, and Nagasaki University, Japan.

### Viral RNA extraction, G and P genotyping, and electropherotyping

Viral RNA was extracted from 140 µL of supernatant obtained from 10% stool suspension (w/v) using a QIAamp Viral RNA Mini Kit (QIAGEN Sciences, Germantown, MD, USA) according to the manufacturer’s instructions. G and P genotyping was done by reverse-transcription PCR by using the primers designed by Gouvea et al. [13] and Gunasena et al. [14]. Genomic RNAs were separated for 16 hours at a constant current of 8 mA on a 10% polyacrylamide gel, and the electropherotype of each strain was determined after staining with silver nitrate as described previously [15].

### Whole-genome amplification and sequencing

Based on the results of the G and P genotyping combined with electropherotyping, five strains were selected for investigation of the whole genome. These included two G1P[8] strains with short RNA migration patterns (SP026 and SP071) and three G2P[4] strains with short RNA migration patterns (SP015, SP108, and SP355). The VP7, VP4 and NSP4 genes of two G1P[8] strains with long RNA migration patterns (SP110 and SP118) were sequenced. An AcessQuick Kit (Promega Corporation, Madison, WI, USA) was used with the gene-specific end primer pairs described previously [3, 16] to generate cDNAs and the full-length amplicons for the 11 genes of SP015, SP026, SP071 and SP108. For strain SP355, the SuperScript III first-strand synthesis system for reverse transcription PCR (Invitrogen, Carlsbad, CA, USA) was used with random hexamers (Invitrogen) to generate the cDNAs, which were then amplified using the GoTaq Green Master Mix System (Promega Corporation) with gene-specific end primer pairs that allowed the generation of full-length amplicons [3, 16]. PrimeSTAR GXL DNA Polymerase (Takara Bio, Inc., Shiga, Japan) was used together with primers designed by Fujii et al. [16] to amplify larger genes that could not be amplified previously.

The amplified full-length genes were purified using an ExoSAP-IT purification kit (USB products, Cleveland, OH, USA) and sequenced from end to end in both the forward and reverse directions by the fluorescent dideoxy chain termination chemistry using a Big Dye Terminator Cycle Sequencing Ready Reaction Kit v3.1 (Applied Biosystems, Foster City, CA, USA). Nucleotide sequence reads were obtained with the aid of an ABI-PRISM 3730 Genetic Analyzer (Applied Biosystems).

### Sequence and phylogenetic analyses

Nucleotide sequences were aligned using the SeqMan programme in the Lasergene 11 software package (DNASTAR, Inc. Madison, WI, USA). The genotype of each genome segment was determined by using the RotaC 2.0 automated genotyping tool for RVA [17].

Multiple sequence alignment was carried out using the MUSCLE programme, and the genetic distances were calculated by the p-distance method implemented in MEGA ver. 6.06 [18]. Nucleotide substitution model testing was carried out in MEGA ver. 6.06, and the best-fit evolutionary model for each gene was selected based on the lowest Bayesian information criterion score. Maximum-likelihood phylogenetic trees were constructed using MEGA ver. 6.06 [18]. Trees were analysed by bootstrapping with 1000 replicates, and inferred by using the general time-reversible model (GTR) with gamma distribution (G) and invariant sites (I) for VP3; GTR+ I for VP1 and VP2; the Tamura-Nei model (TN93) + I for NSP3; the Tamura 3-parameter (T92) +G for VP7, VP4, VP6, NSP2, NSP4 and NSP5; and T92+I for NSP1 [19].

### Nucleotide sequence accession numbers

The nucleotide sequences were deposited under the accession numbers LC066147-LC066196, and LC174963-LC174973, and the lengths of these sequences are listed in Supplementary Table 1.

## Results

### Distribution of genotypes, identification of electropherotypes, and selection of strains for sequencing

Out of the 382 faecal specimens that were collected from children less than two years of age who were hospitalised for acute diarrhoea, RVA was detected in 141 (37%) specimens. Of these, 72 (51%) specimens were genotyped as G1P[8] and 50 (35%) as G2P[4]. Thus, G1P[8] and G2P[4] were co-dominant, together accounting for 86% of the rotavirus-positive specimens detected during the study period.

**Fig. 1:**
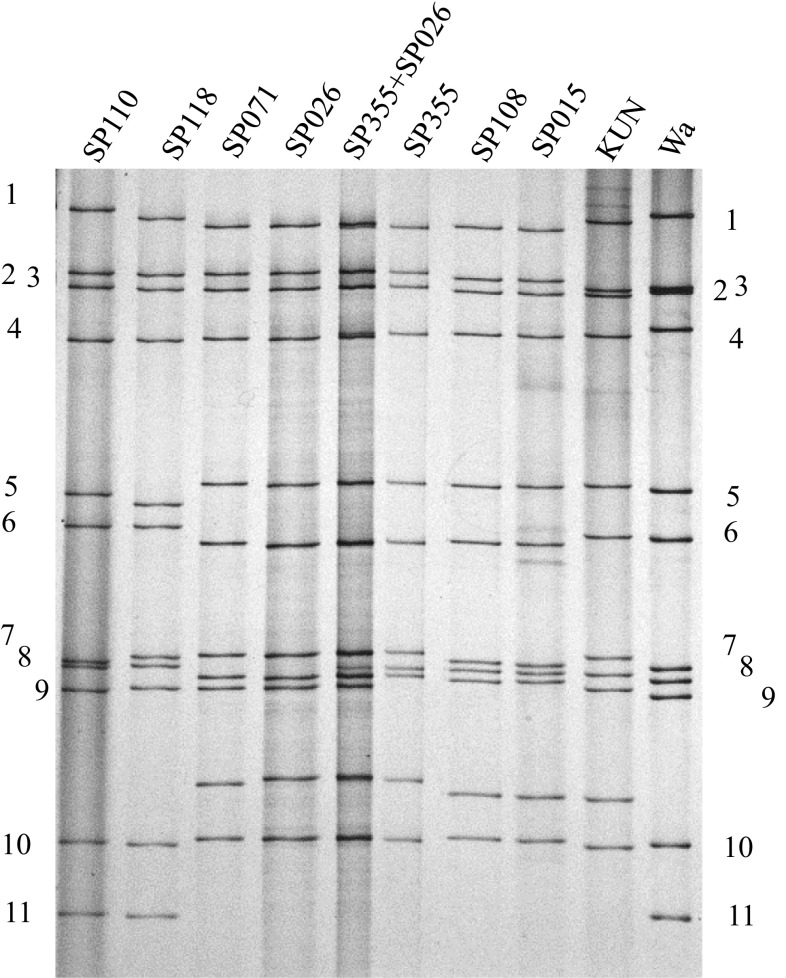
Electrophoretic migration pattern of genomic double-stranded RNAs of Vietnamese RVA strains detected between November 2012 and June 2013 alongside Wa (long RNA migration pattern) and KUN (short RNA migration pattern) as controls on a 10% polyacrylamide gel stained with silver nitrate. Strain names are indicated at the top of each lane on the gel, while numbers on both sides represent the genome segment number. SP110 and SP118 were the most and the second most dominant G1P[8] strains, respectively, exhibiting long RNA migration patterns. SP071 and SP026 are virtually identical G1P[8] strains with short RNA migration patterns. Note the slight migration difference in the position of the 10th genome segments. SP355+SP026: co-electrophoresis of the RNAs from the two strains in the same lane. Note that nine genome segments of SP355 and SP026 co-migrated. SP108 and SP015 are the representatives of the predominant G2P[4] strains selected for whole-genome sequencing. Wa and KUN represent G1P[8] and G2P[4], respectively

When the genomic RNAs of the 72 G1P[8] RVA specimens were separated on polyacrylamide gels, 49 (68%) samples exhibited long RNA migration patterns, and 20 (28%) exhibited short RNA migration patterns. Of those with long RNA migration patterns, the electropherotypes of 33 specimens were identical to that of SP110, and five were identical to that of SP118 (Fig. 1). Of the 20 specimens exhibiting short RNA migration patterns, the electropherotypes were virtually identical, with 10 specimens identical to SP026 and the remaining 10 identical to SP071 (Fig. 1 and Supplementary Fig. 1). Thus, both SP026 and SP071 were selected for whole-genome sequencing, and SP110 and SP118 were selected for sequencing of the VP7, VP4 and NSP4 genes.

Out of 50 G2P[4] RVA specimens, the electropherotypes of 44 (88%) were identical, from which SP108 and SP015 were selected for whole-genome sequencing (Fig. 1). While unique in its electropherotype, nine genome segments of SP355 and those of SP026 co-migrated upon co-electrophoresis (Fig. 1). Thus, SP355 was selected for whole-genome sequencing.

### Determination of the nearly complete genome sequences and genotype constellations of Vietnamese G1P[8] strains exhibiting short RNA migration patterns (SP026 and SP071)

Nearly-complete sequences of the 11 genes of SP026 and SP071 were determined (Supplementary Table 1), and their genotype constellations were found to be G1-P[8]-I2-R2-C2-M2-A2-N2-T2-E2-H2. Thus, both strains were generated as the result of genetic reassortment between a G1P[8] strain and a DS-1-like G2P[4] strain; hence, they are G1P[8] double-gene reassortants.

When the nucleotide sequences of the 11 genes of SP026 and SP071 were compared, 10 of them were 99.9-100% identical and the NSP5 gene (the 10th genome segment) differed slightly more, with 99.6% sequence identity (Table 1). This observation was in good agreement with the identical electropherotypes including the minimally observable migration difference in the 10th genome segments, which encode NSP5 (Fig. 1). This indicated that the Vietnamese G1P[8] double-gene reassortant strains were of clonal origin.

**Table 1:** Nucleotide sequence identities (%) between the Vietnamese double-gene reassortant strain SP071 and designated strains in each genome segment

Strain	VP7	VP4	VP6	VP1	VP2	VP3	NSP1	NSP2	NSP3	NSP4	NSP5
**SP026/VNM/2012/G1P[8]**	99.9	100.0	99.9	99.9	100.0	100.0	99.9	99.9	99.9	100.0	99.6
**SP355/VNM/2013/G2P[4]**	67.3	85.7	99.3	99.6	99.7	99.5	99.4	99.7	100.0	99.6	99.6
**SP015/VNM/2012/G2P[4]**	73.2	87.0	96.5	94.5	98.3	99.3	96.7	99.2	97.1	99.2	98.3
**SP108/VNM/2012/G2P[4]**	73.1	87.0	96.7	94.6	98.3	99.2	96.7	99.3	97.3	99.2	98.3
**SP110/VNM/2012/G1P[8]**	99.1	98.0	nd	nd	nd	nd	nd	nd	nd	84.5	nd
**SP118/VNM/2013/G1P[8]**	98.9	97.2	nd	nd	nd	nd	nd	nd	nd	84.1	nd
BX5/CHN/2007/G1P[8]	99.5	98.4	na	na	na	na	na	na	na	na	na
R1604/CHN/2011/G3P[8]	72.5	99.8	I1	R1	C1	M1	A1	N1	T1	E1	H1
YR049/JPN/2012/G1P[8]	95.7	98.5	98.5	99.2	99.3	97.5	99.1	97.2	99.3	97.3	98.8
SSKT-41/THA/2013/G1[8]	95.4	98.8	98.7	99.2	99.2	97.5	99.0	97.0	98.9	97.0	99.0

Strain names whose sequences were determined in this study are in bold. *nd* not done; *na* not available

### Comparison of Vietnamese G1P[8] strains exhibiting short RNA migration patterns with locally circulating G2P[4] strains

**Fig. 2:**
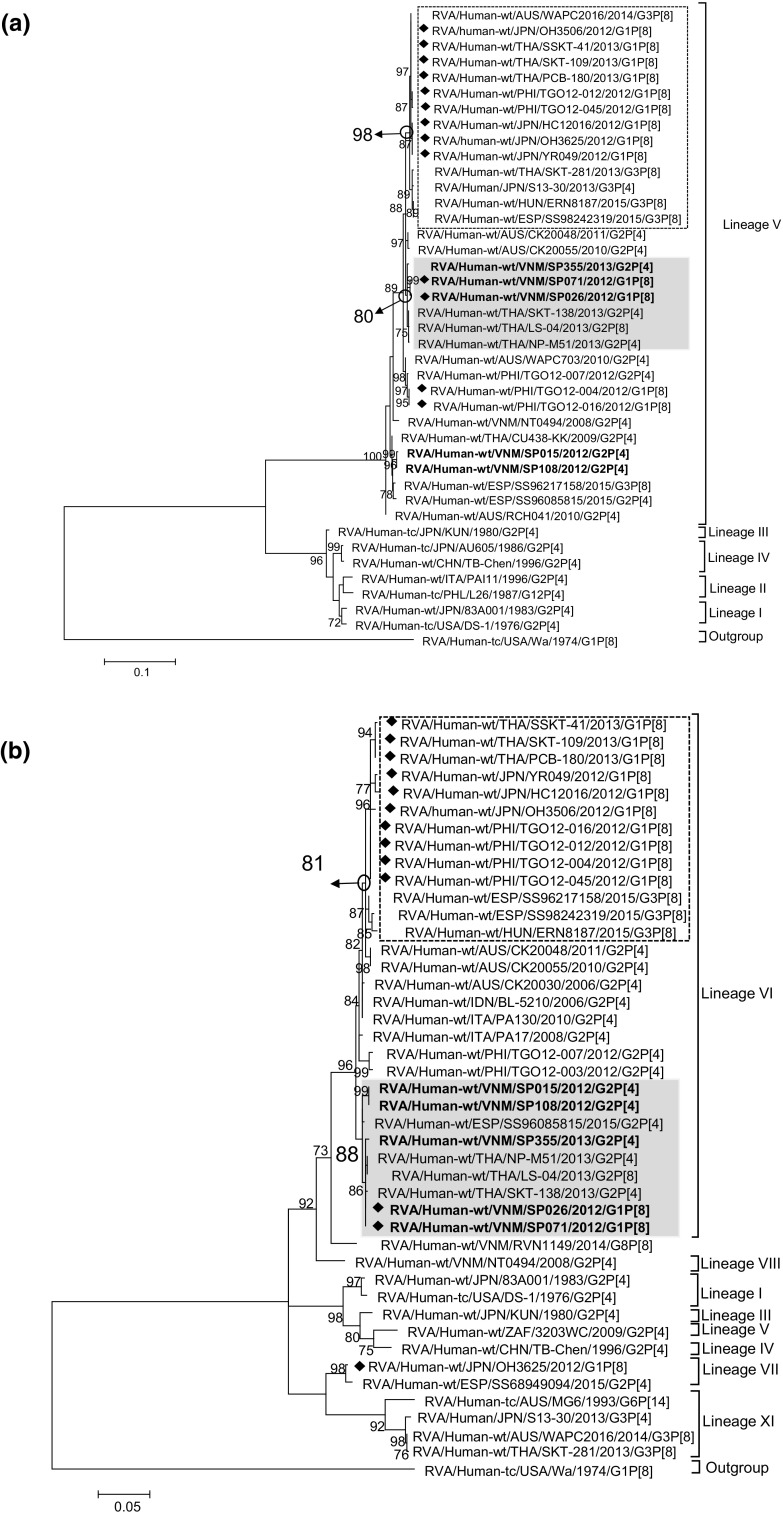

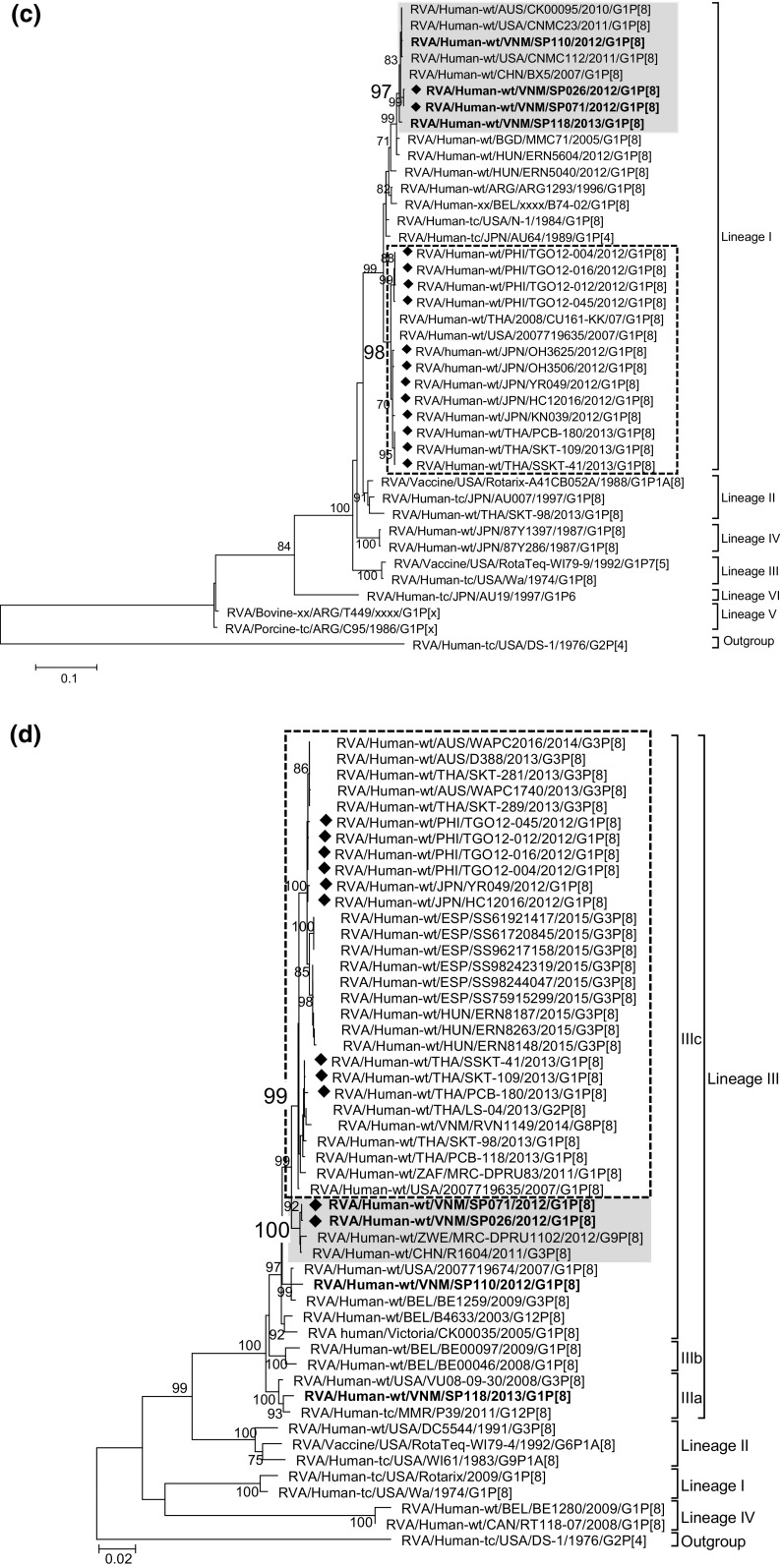
Maximum-likelihood phylogenetic trees drawn for the (a) VP6 gene of I2 genotype, (b) NSP4 gene of E2 genotype, (c) VP7 gene of G1 genotype, and (d) VP4 gene of P[8] genotype. Vietnamese strains sequenced in this study are highlighted in bold. The diamond to the left of the strain name indicates a G1P[8] double-gene reassortant strain. The cluster that contains the Vietnamese double-gene reassortants (SP026/SP071) and G2P[4] strains from Vietnam (SP355) and Thailand (SKT-138 and NP-M51) are shaded in grey. The cluster that contains G1P[8] double-gene reassortant strains detected in Japan and Thailand is enclosed in dotted lines. Bootstrap values that are equal to or larger than 70% after 1000 replicate trials are indicated at each node. The scale bar at the bottom of the tree indicates the genetic distance expressed as nucleotide substitutions per site

High nucleotide sequence identities between SP355 and SP026/SP071 were observed for all internal capsid and non-structural protein genes, with the VP6 gene showing the lowest level of nucleotide sequence identity of 99.2% (SP355 vs SP026) and the NSP3 gene showing the highest identity of 100% (SP355 vs. SP071) (Table 1). When a maximum-likelihood tree was drawn for the VP6 gene, a cluster was identified within lineage V with 80% bootstrap support that comprised the sequences of the double-gene reassortant strains (SP071 and SP026) and those of ordinary G2P[4] strains circulating in Vietnam (SP355) and Thailand (SKT-138 and NP-M51) (shaded in grey in Fig. 2a). Similarly, in the NSP4 tree, a cluster was identified within lineage VI with 88% bootstrap support that comprised the sequences of SP071 and SP026 as well as SP355, SKT-138 and NP-M51 (shaded in grey in Fig. 2b). The same clustering relationship was observed in the trees drawn for the rest of the internal capsid and non-structural protein genes without an exception and with high bootstrap support (Supplementary Fig. 2a-g). Thus, SP026/SP071 and SP355 possessed a virtually identical set of nine genes encoding the internal capsid and non-structural proteins. The observation that these clusters always contained G2P[4] strains detected in Thailand (SKT-138 and NP-M51) indicated that SP355-like strains circulated beyond a limited location in Vietnam and were present in wider geographic locations.

On the other hand, the predominant G2P[4] strains (SP015 and SP108) detected during the study period possessed moderately high sequence identities of 99.2-99.3% only for the genes encoding VP3, NSP2 and NSP4 (Table 1). In the phylogenetic trees drawn for these three genes, the sequences of SP015 and SP108 belonged to the cluster that comprised those of the Vietnamese G1P[8] double-gene reassortant strains SP026 and SP071 and the Vietnamese and Thai G2P[4] strains SP355, SKT-138 and NP-M51 (Supplementary Fig. 2 and e, and Fig. 2b). However, the rest of the internal capsid and non-structural protein genes of SP015 and SP108 showed lower nucleotide sequence identity (97.1-98.3%) (Table 1), and they belonged to a cluster different from the double-gene reassortant strains (Fig. 2a, Supplementary Fig. 2a, b, d, f, and g).

### Search for the origin of the outer-capsid genes of the Vietnamese double-gene reassortant strains

The VP7 gene of the G1P[8] double-gene reassortant strains (SP026 and SP071) showed moderately high sequence identities of 99.1% and 98.9% to those of the locally circulating G1P[8] strains SP110 and SP118, respectively, but this level of sequence identity was not observed for the VP4 gene (98.0% and 97.2%, respectively) (Table 1). To find RVA strains that might possess outer-capsid genes that are more similar to those of SP026 and SP071, the GenBank database was searched using the Basic Local Alignment Search Tool with SP071 as the query sequence. The search found that a Chinese strain (RVA/Human-wt/CHN/BX5/2007/G1P[8]) was 99.5% identical to SP071 in the VP7 gene (Table 1), and another Chinese strain (RVA/Human-wt/CHN/R1604/2011/G3P[8]) was 99.8% identical in the VP4 gene (Table 1).

Maximum-likelihood trees were drawn for the G1 VP7 and P[8] VP4 genes (Fig. 2c and d). Within lineage I of the G1VP7 tree, a cluster was identified with 97% bootstrap support that comprised SP071, SP026, and Chinese BX5 as well as Vietnamese SP110 and SP118 (shaded in grey Fig. 2c). Notably, however, this cluster was distinct from the cluster that comprised the Japanese and Thai G1P[8] double-gene reassortant strains (Fig. 2c).

In the VP4 tree, SP071 and SP026 clustered together with the Chinese G3P[8] strain R1604 with 100% bootstrap support (shaded in grey in Fig. 2d), whereas SP110 and SP118 belonged to different clusters, although all of them were within P[8] lineage III (Fig. 2d). In addition, the VP4 genes of Japanese and Thai double-gene reassortant strains clustered with 99% bootstrap support with those of G3P[8] double-gene reassortant strains detected in Thailand, Australia, Spain and Hungary, and this cluster was distinct from the cluster comprising SP071 and SP026 (Fig. 2d).

### Relationship between the internal capsid and non-structural protein genes of the Vietnamese and Japanese/Thai G1P[8] double-gene reassortant strains

In the trees drawn for each of the internal capsid and non-structural protein genes of the Vietnamese and Japanese/Thai double-gene reassortant strains, the cluster comprising the Vietnamese double-gene reassortant strains was clearly distinguished from the cluster comprising the Japanese/Thai double-gene reassortant strains, since each cluster was supported by a high bootstrap value (shaded in grey and enclosed in dotted lines in Fig. 2a and b and Supplementary Fig. 2a-g), providing evidence for independent reassortment events of the Vietnamese and Japanese/Thai strains.

### Comparison of the mean severity scores between children infected with ordinary G1P[8] strains and those infected with G1P[8] double-gene reassortant strains

**Fig. 3:**
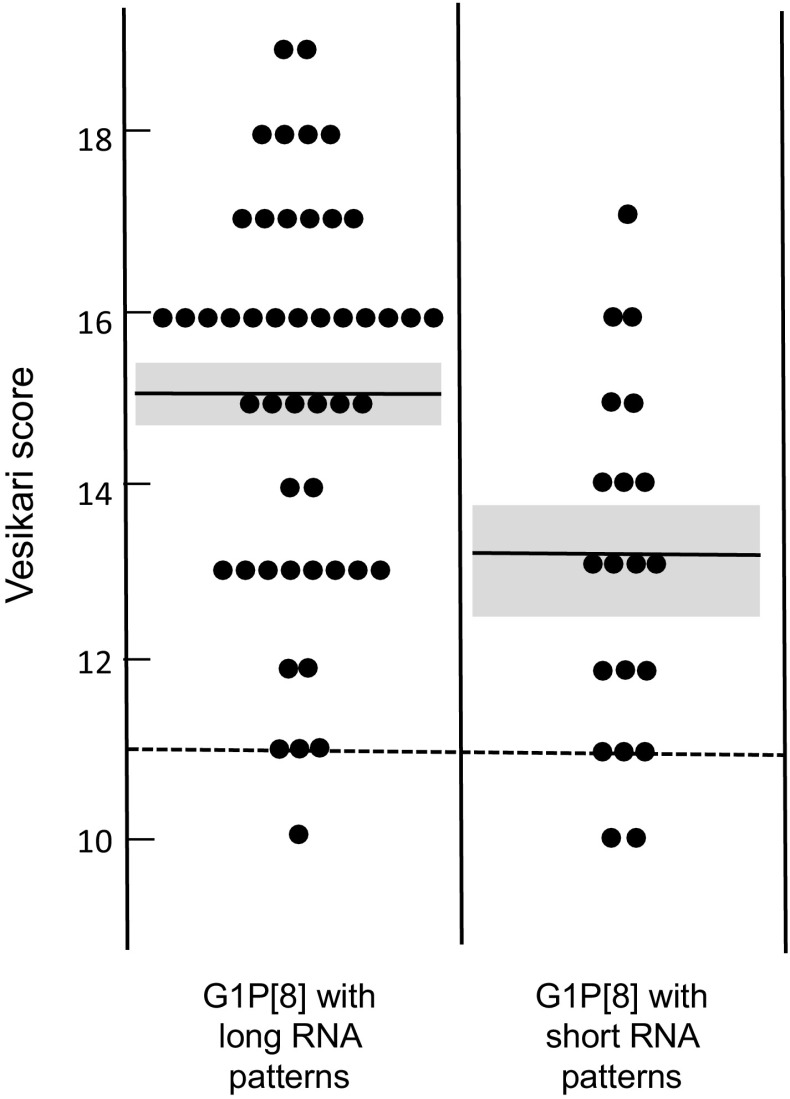
The distribution of severity scores for the patients infected with ordinary G1P[8] strains (long RNA migration patterns) and the patients infected with double-gene reassortant strains (short RNA migration patterns). Horizontal bars show the mean severity scores, and 95% confidence intervals are shaded in grey. The dotted line shows the minimum score for severe diarrhoea

To assess whether G1P[8] double-gene reassortant strains caused more-severe disease than ordinary G1P[8] strains, the severity scores were calculated according to Vesikari’s 20-point scale by using the clinical information collected at the time of hospitalisation. The distribution of severity scores for each group of patients is shown in Fig. 3. The mean severity scores calculated for the patients with ordinary G1P[8] strains (n = 49) and double-gene reassortant strains (n = 20) were 15.1 (SD = 2.23, 95% confidence interval [CI]: 14.7-15.5) and 13.1 (SD = 2.02, 95%CI: 12.5-13.7), respectively. As diarrhoea with a Vesikari score ≥11 is defined as severe diarrhoea, both groups of patients experienced severe diarrhoea. Statistically, however, the patients infected with ordinary G1P[8] strains were shown to experience slightly more severe diarrhoea than those infected with double-gene reassortant strains (Student’s *t*-test, two-tailed, *P* = 0.0010).

## Discussion

The identification of G1P[8] double-gene reassortant strains in Vietnam in the 2012/2013 rotavirus season is reminiscent of the DS-1-like G1P[8] double-gene reassortant strains emerging in one rotavirus season earlier in Japan [7–9] and the other strains emerging in the same rotavirus season in Thailand [10]. In Thailand, the detection was limited to three sporadic cases accounting for 0.4% of the circulating strains [10]. On the other hand, in Japan, they caused a country-wide spread and accounted for 31-62% of the strains, depending on the region [9]. Based on high nucleotide sequence identities of 98.5-99.7% and co-clustering topology in phylogenetic trees for all 11 genes, Komoto et al. [10] hypothesised that Japanese and Thai double-gene reassortant strains originated from a recent common ancestor.

While the determination of the whole genotype constellation showed that all of these DS-1-like G1P[8] strains were generated by genetic reassortment, previous studies failed to identify the G2P[4] strain that donated the DS-1-like background of the double-gene reassortant strains. In this study, determination of the whole genome sequence and phylogenetic analysis showed that the internal-capsid and non-structural protein genes of SP355 were 99.3-100% identical to those of SP026/SP071, and they clustered together in the same lineage in the phylogenetic trees.

With respect to the origin of the outer-capsid genes of the Vietnamese double-gene reassortant strains, two Chinese strains that had the highest nucleotide sequence identities (99.5% and 99.8% identical to the Vietnamese VP7 and VP4 genes, respectively) clustered with SP026 and SP071 with very high bootstrap support. The G1P[8] strains co-circulating during the study period belonged to the same cluster with the SP026/SP071 in the VP7 tree, but not in the VP4 tree. Nevertheless, when the geographical closeness of Vietnam and China and our limited strain sampling were taken into consideration, it is likely that locally circulating G1P[8] strains in Vietnam donated their VP7 and VP4 genes to the SP355-like strains, resulting in the emergence of the double-gene reassortants in Vietnam.

The results obtained in this study led us to hypothesise that the Vietnamese G1P[8] strains were most likely to have been generated in Vietnam through the genetic reassortment events in which a locally circulating G2P[4] strain acquired the outer-capsid genes from co-circulating G1 and P[8] strains. The generation of these double-gene reassortant strains is unlikely to be attributable to the events in which the Japanese and Thai double-gene reassortants were generated on the following grounds: First, the Vietnamese double-gene reassortant strains always clustered together with a locally-circulating G2P[4] strain, and this cluster, which was supported by a high bootstrap value, was clearly distinguished from the cluster comprising the Japanese/Thai double-gene reassortant strains. The Vietnamese G2P[4] strain (SP355) together with Thai G2P[4] strains (SKT138 and NP-M51) never clustered with the Japanese/Thai double-gene reassortant strains in any of the internal-capsid and non-structural protein genes, indicating that they were unlikely to be the donor strains that provided the backbone of the Japanese/Thai double-gene reassortant strains. Second, the outer-capsid genes that clustered together with those of the Vietnamese and Japanese/Thai double-gene reassortant strains were from different strains: the Chinese G1P[8] strain BX5 and G3P[8] strain R1604 for the former, and the American G1P[8] strain 2007719635 for the latter.

While the further evolution of any of the G1P[8] double-gene reassortant strains from Japan and Thailand is unclear, the emergence of G3P[8] double-gene reassortant strains in Australia [20], Thailand [21], and Spain [22] deserves mention because they also possessed the DS-1-like internal-capsid and non-structural protein genes. Sporadically detected Thai G3P[8] double-gene reassortant strains, although co-circulating with the G1P[8] double-gene reassortant strains in the same rotavirus season, were described as having their nine genes having originated from G1P[8] double-gene reassortant strains [21]. The G3P[8] strains that caused a local outbreak in the Basque country in northern Spain were hypothesised to have originated from the G3P[8] strains that had appeared earlier in the Asian Pacific region [22].

In Vietnam, bovine-like G8P[8] strains possessing a DS-1-like backbone emerged in 2014 and became dominant in 2015 [23], but their backbone genes as well as the P[8] VP4 gene were unlikely to have been direct descendants of the genes of the Vietnamese G1P[8] double-gene reassortant strains described in this study.

Few studies have addressed the question of whether recently emerging strains with unusual genotype constellations, such as G1P[8] double-gene reassortant strains, cause more-severe disease in children than ordinary RVA strains. In this study, it was shown that children infected with Vietnamese G1P[8] double-gene reassortant strains experienced severe diarrhoea (as the mean Vesikari score of 13.1 was categorised as severe diarrhoea) but slightly less-severe diarrhoea than those children infected with ordinary G1P[8] strains (Fig. 3). Since this study did not examine other enteric pathogens that might have co-infected the children under investigation, the observed difference may not simply be ascribed to the difference in the pathogenic potential of the strains compared. Thus, it can be concluded that the disease caused by G1P[8] double-gene reassortant strains was not more severe than that caused by common G1P[8] strains.

In conclusion, this study showed that apparently clonal G1P[8] double-gene reassortant strains emerged in Hanoi, Vietnam and accounted for 14% of the RVA-positive specimens recovered from infants and young children hospitalised for severe diarrhoea during the 2012/2013 rotavirus season. Whole-genome analysis showed that these Vietnamese strains were generated by genetic reassortment events in which a locally circulating G2P[4] strain acquired the VP7 and VP4 genes from strains similar to Chinese G1P[8] and G3P[8] strains, respectively. Despite the similarity in their emergence in time (2012/2013) and place (the Western Pacific region), the Vietnamese and Japanese/Thai G1P[8] double-gene reassortant strains were generated by different combinations of parental strains.

## Electronic supplementary material

Below is the link to the electronic supplementary material.

Supplementary material 1 (DOCX 14 kb)

Supplementary material 2 (PPT 278 kb)

Supplementary material 3 (PPT 613 kb)
