# Supplementary material for: Evolution of DS-1-like G1P[8] double-gene reassortant rotavirus A strains causing gastroenteritis in children in Vietnam in 2012/2013
Source: Arch Virol. 2016 Nov 23;162(3):739–48. doi: 10.1007/s00705-016-3155-6 (PMC5329091; doi:10.1007/s00705-016-3155-6)
Supplement: Supplementary file 1 — Supplementary material 1 (DOCX 14 kb) [file 705_2016_3155_MOESM1_ESM.docx]

Supplementary Table 1 Accession numbers for and the nucleotide positions of the genes of rotavirus strains determined in this study.

|  | **VP7** | **VP4** | **VP6** | **VP1** | **VP2** | **VP3** | **NSP1** | **NSP2** | **NSP3** | **NSP4** | **NSP5** |
| --- | --- | --- | --- | --- | --- | --- | --- | --- | --- | --- | --- |
| **SP026** | LC066147 | LC066148 | LC066149 | LC066150 | LC066151 | LC066152 | LC066153 | LC066154 | LC066155 | LC066156 | LC066157 |
|  | 29-1035 | 13-2338 | 21-1339 | 24-3279 | 28-2659 | 21-2570 | 25-1548 | 20-1041 | 22-1044 | 22-731 | 20-799 |
| **SP071** | LC066158 | LC066159 | LC066160 | LC066161 | LC066162 | LC066163 | LC066164 | LC066165 | LC066166 | LC066167 | LC066168 |
|  | 29-1035 | 13-2338 | 21-1339 | 24-3279 | 28-2659 | 21-2570 | 25-1548 | 20-1041 | 22-1044 | 22-731 | 20-799 |
| **SP015** | LC066169 | LC066170 | LC066171 | LC066172 | LC066173 | LC066174 | LC066175 | LC066176 | LC066177 | LC066178 | LC066179 |
|  | 29-1035 | 13-2338 | 21-1339 | 24-3279 | 28-2659 | 21-2570 | 25-1548 | 20-1041 | 22-1044 | 22-731 | 20-799 |
| **SP108** | LC066180 | LC066181 | LC066182 | LC066183 | LC066184 | LC066185 | LC066186 | LC066187 | LC066188 | LC066189 | LC066190 |
|  | 29-1035 | 13-2338 | 21-1339 | 24-3279 | 28-2659 | 21-2570 | 25-1548 | 20-1041 | 22-1044 | 22-731 | 20-799 |
| **SP355** | LC174963 | LC174964 | LC174965 | LC174966 | LC174967 | LC174968 | LC174969 | LC174970 | LC174971 | LC174972 | LC174973 |
|  | 23-1039 | 21-2347 | 21-1339 | 23-3276 | 28-2659 | 25-2565 | 25-1548 | 22-1035 | 22-1044 | 22-731 | 25-796 |
| **SP110** | LC066191 | LC066192 |  |  |  |  |  |  |  | LC066193 |  |
|  | 29-1035 | 13-2337 |  |  |  |  |  |  |  | 22-730 |  |
| **SP118** | LC066194 | LC066195 |  |  |  |  |  |  |  | LC066196 |  |
|  | 29-1035 | 13-2337 |  |  |  |  |  |  |  | 22-730 |  |
