# Supplementary material for: Evolution of DS-1-like G1P[8] double-gene reassortant rotavirus A strains causing gastroenteritis in children in Vietnam in 2012/2013
Source: Arch Virol. 2016 Nov 23;162(3):739–48. doi: 10.1007/s00705-016-3155-6 (PMC5329091; doi:10.1007/s00705-016-3155-6)
Supplement: Supplementary file 2 — Supplementary material 2 (PPT 278 kb) [file 705_2016_3155_MOESM2_ESM.ppt]

## Slide 1
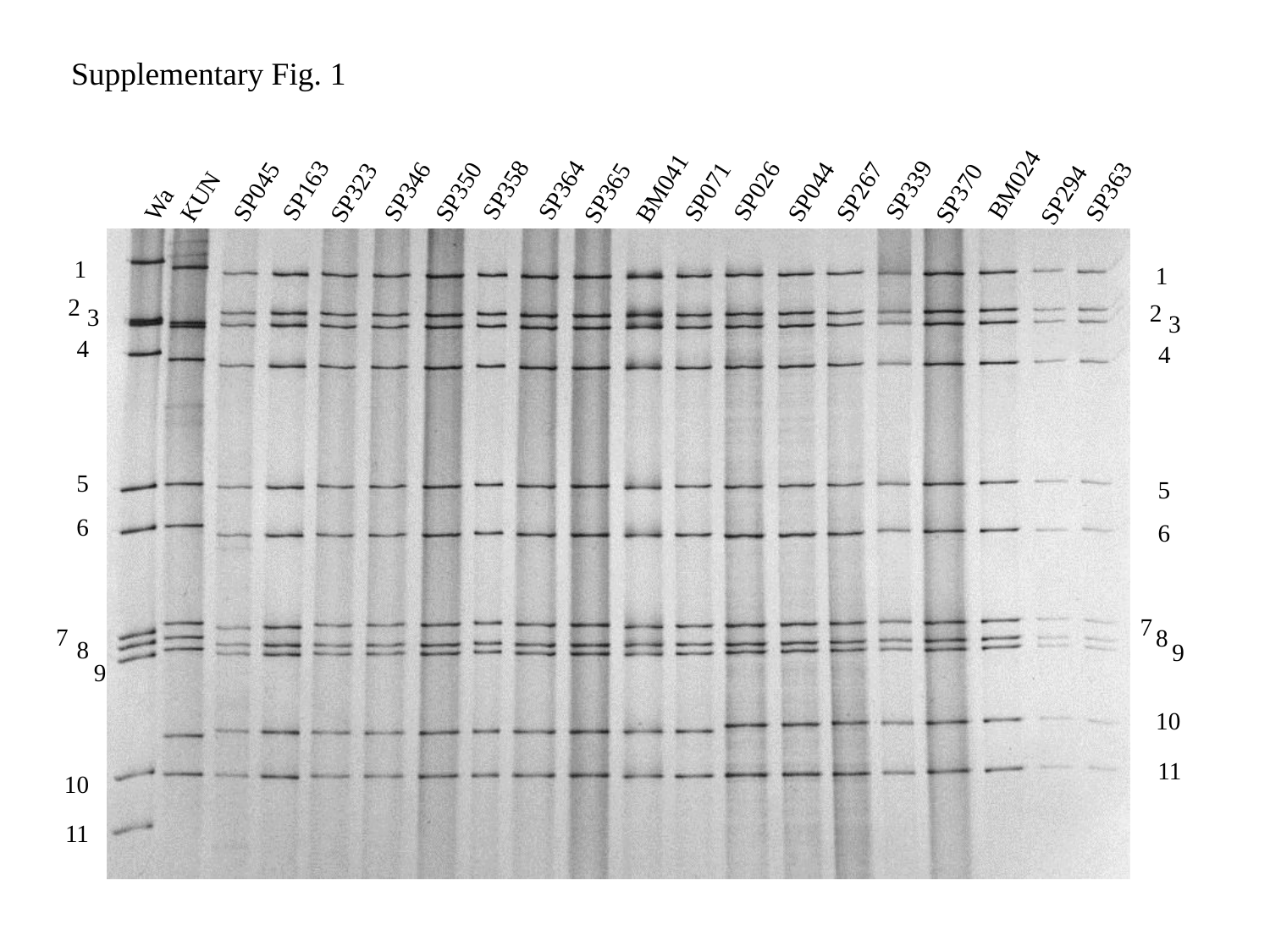

Supplementary Fig. 1
SP163
SP339
SP026
SP045
SP071
SP267
SP044
SP323
BM041
SP365
SP370
SP364
SP358
BM024
SP363
SP294
SP350
SP346
KUN
Wa
1
1
2
2
3
3
4
4
5
5
6
6
7
7
8
8
9
9
10
11
10
11

## Slide 2
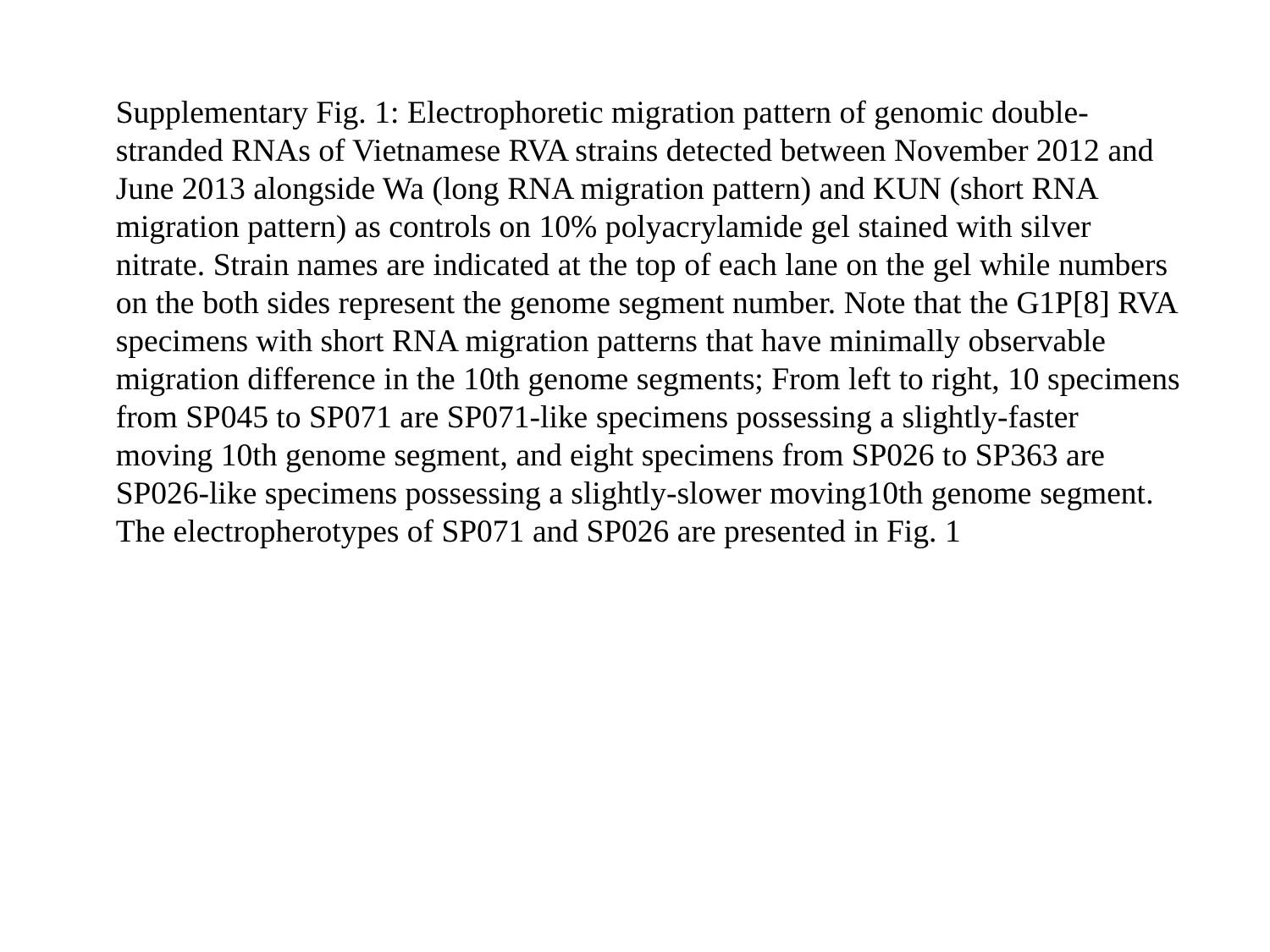

Supplementary Fig. 1: Electrophoretic migration pattern of genomic double-stranded RNAs of Vietnamese RVA strains detected between November 2012 and June 2013 alongside Wa (long RNA migration pattern) and KUN (short RNA migration pattern) as controls on 10% polyacrylamide gel stained with silver nitrate. Strain names are indicated at the top of each lane on the gel while numbers on the both sides represent the genome segment number. Note that the G1P[8] RVA specimens with short RNA migration patterns that have minimally observable migration difference in the 10th genome segments; From left to right, 10 specimens from SP045 to SP071 are SP071-like specimens possessing a slightly-faster moving 10th genome segment, and eight specimens from SP026 to SP363 are SP026-like specimens possessing a slightly-slower moving10th genome segment. The electropherotypes of SP071 and SP026 are presented in Fig. 1
