# Supplementary material for: Evolution of DS-1-like G1P[8] double-gene reassortant rotavirus A strains causing gastroenteritis in children in Vietnam in 2012/2013
Source: Arch Virol. 2016 Nov 23;162(3):739–48. doi: 10.1007/s00705-016-3155-6 (PMC5329091; doi:10.1007/s00705-016-3155-6)
Supplement: Supplementary file 3 — Supplementary material 3 (PPT 613 kb) [file 705_2016_3155_MOESM3_ESM.ppt]

## Slide 1
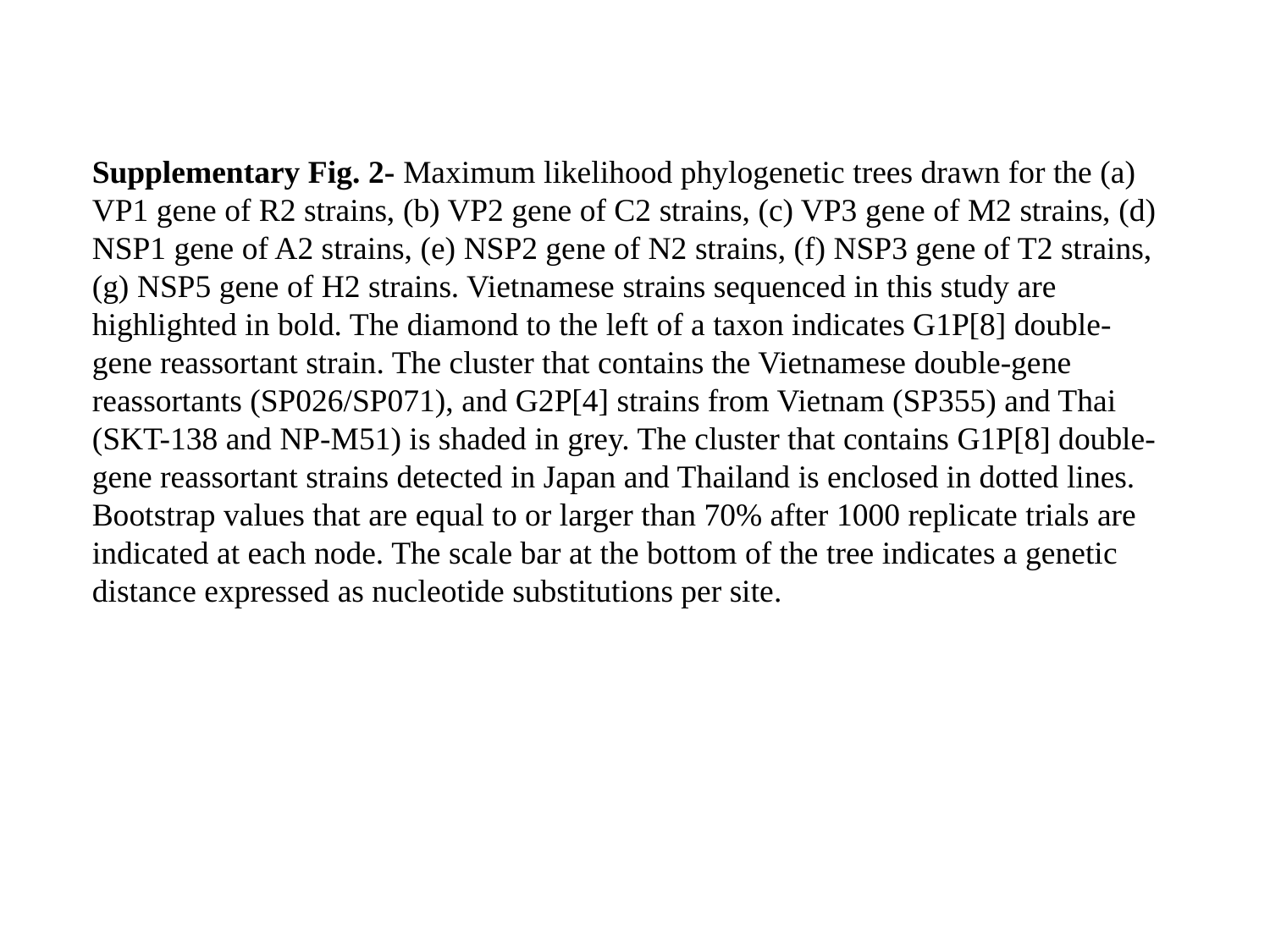

Supplementary Fig. 2- Maximum likelihood phylogenetic trees drawn for the (a) VP1 gene of R2 strains, (b) VP2 gene of C2 strains, (c) VP3 gene of M2 strains, (d) NSP1 gene of A2 strains, (e) NSP2 gene of N2 strains, (f) NSP3 gene of T2 strains, (g) NSP5 gene of H2 strains. Vietnamese strains sequenced in this study are highlighted in bold. The diamond to the left of a taxon indicates G1P[8] double-gene reassortant strain. The cluster that contains the Vietnamese double-gene reassortants (SP026/SP071), and G2P[4] strains from Vietnam (SP355) and Thai (SKT-138 and NP-M51) is shaded in grey. The cluster that contains G1P[8] double-gene reassortant strains detected in Japan and Thailand is enclosed in dotted lines. Bootstrap values that are equal to or larger than 70% after 1000 replicate trials are indicated at each node. The scale bar at the bottom of the tree indicates a genetic distance expressed as nucleotide substitutions per site.

## Slide 2
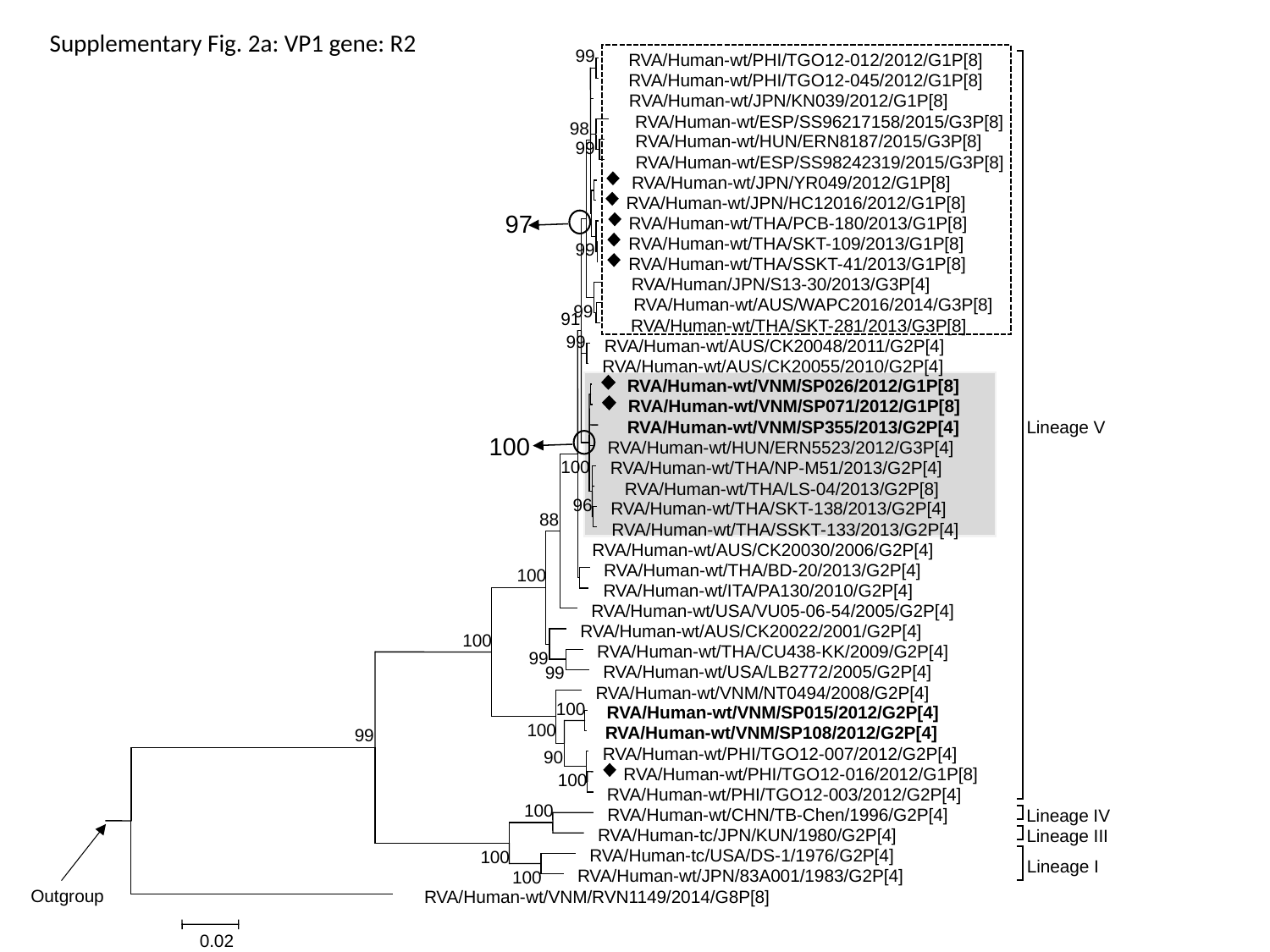

Supplementary Fig. 2a: VP1 gene: R2
99
 RVA/Human-wt/PHI/TGO12-012/2012/G1P[8]
 RVA/Human-wt/PHI/TGO12-045/2012/G1P[8]
 RVA/Human-wt/JPN/KN039/2012/G1P[8]
 RVA/Human-wt/ESP/SS96217158/2015/G3P[8]
98
 RVA/Human-wt/HUN/ERN8187/2015/G3P[8]
99
 RVA/Human-wt/ESP/SS98242319/2015/G3P[8]
 RVA/Human-wt/JPN/YR049/2012/G1P[8]
 RVA/Human-wt/JPN/HC12016/2012/G1P[8]
97
 RVA/Human-wt/THA/PCB-180/2013/G1P[8]
 RVA/Human-wt/THA/SKT-109/2013/G1P[8]
99
 RVA/Human-wt/THA/SSKT-41/2013/G1P[8]
 RVA/Human/JPN/S13-30/2013/G3P[4]
 RVA/Human-wt/AUS/WAPC2016/2014/G3P[8]
99
91
 RVA/Human-wt/THA/SKT-281/2013/G3P[8]
99
 RVA/Human-wt/AUS/CK20048/2011/G2P[4]
 RVA/Human-wt/AUS/CK20055/2010/G2P[4]
 RVA/Human-wt/VNM/SP026/2012/G1P[8]
 RVA/Human-wt/VNM/SP071/2012/G1P[8]
 RVA/Human-wt/VNM/SP355/2013/G2P[4]
Lineage V
100
 RVA/Human-wt/HUN/ERN5523/2012/G3P[4]
100
 RVA/Human-wt/THA/NP-M51/2013/G2P[4]
 RVA/Human-wt/THA/LS-04/2013/G2P[8]
96
 RVA/Human-wt/THA/SKT-138/2013/G2P[4]
88
 RVA/Human-wt/THA/SSKT-133/2013/G2P[4]
 RVA/Human-wt/AUS/CK20030/2006/G2P[4]
 RVA/Human-wt/THA/BD-20/2013/G2P[4]
100
 RVA/Human-wt/ITA/PA130/2010/G2P[4]
 RVA/Human-wt/USA/VU05-06-54/2005/G2P[4]
 RVA/Human-wt/AUS/CK20022/2001/G2P[4]
100
 RVA/Human-wt/THA/CU438-KK/2009/G2P[4]
99
 RVA/Human-wt/USA/LB2772/2005/G2P[4]
99
 RVA/Human-wt/VNM/NT0494/2008/G2P[4]
100
 RVA/Human-wt/VNM/SP015/2012/G2P[4]
100
 RVA/Human-wt/VNM/SP108/2012/G2P[4]
99
 RVA/Human-wt/PHI/TGO12-007/2012/G2P[4]
90
 RVA/Human-wt/PHI/TGO12-016/2012/G1P[8]
100
 RVA/Human-wt/PHI/TGO12-003/2012/G2P[4]
100
 RVA/Human-wt/CHN/TB-Chen/1996/G2P[4]
Lineage IV
 RVA/Human-tc/JPN/KUN/1980/G2P[4]
Lineage III
 RVA/Human-tc/USA/DS-1/1976/G2P[4]
100
Lineage I
 RVA/Human-wt/JPN/83A001/1983/G2P[4]
100
 RVA/Human-wt/VNM/RVN1149/2014/G8P[8]
0.02
Outgroup

## Slide 3
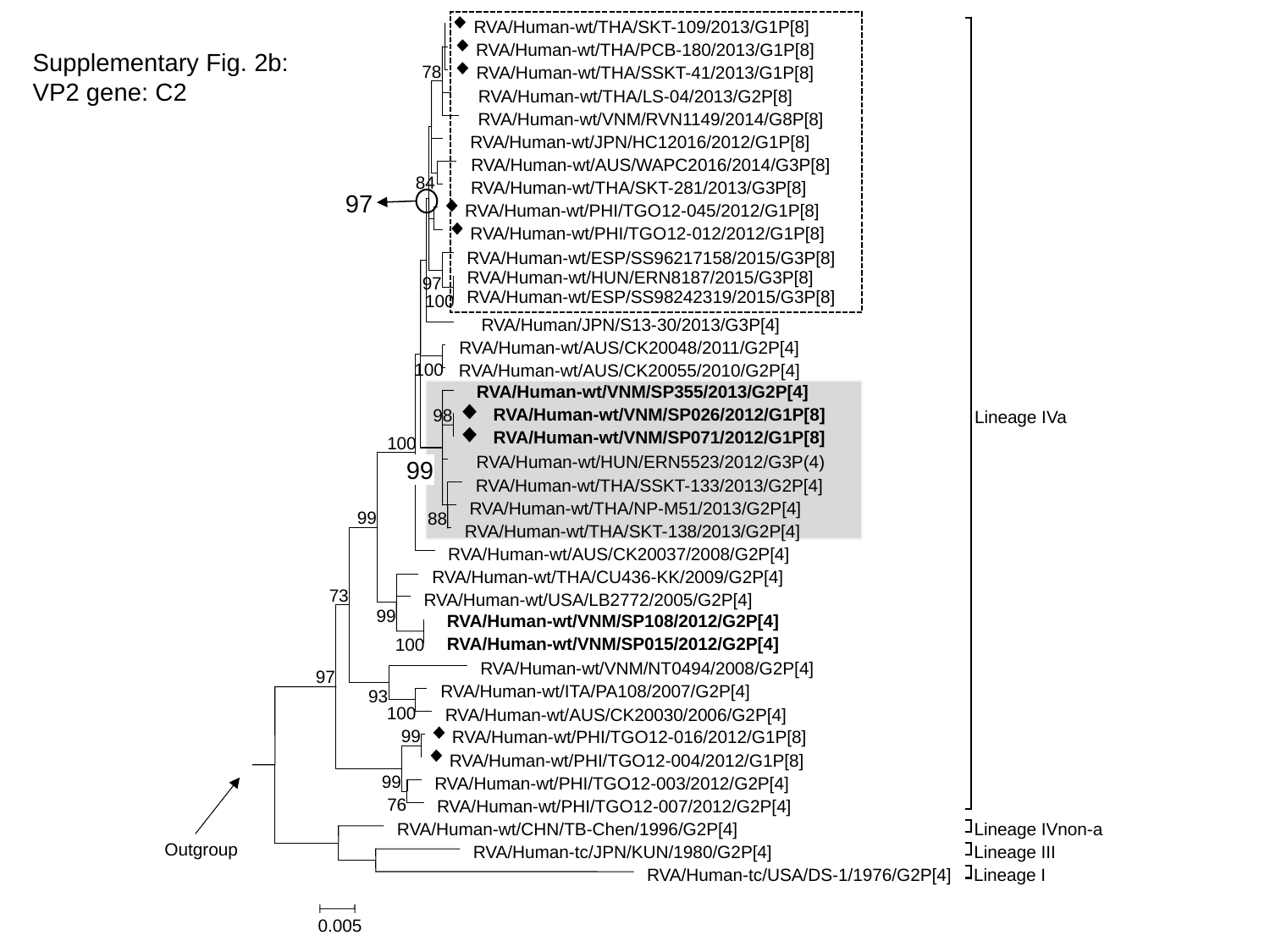

RVA/Human-wt/THA/SKT-109/2013/G1P[8]
 RVA/Human-wt/THA/PCB-180/2013/G1P[8]
78
 RVA/Human-wt/THA/SSKT-41/2013/G1P[8]
 RVA/Human-wt/THA/LS-04/2013/G2P[8]
 RVA/Human-wt/VNM/RVN1149/2014/G8P[8]
 RVA/Human-wt/JPN/HC12016/2012/G1P[8]
 RVA/Human-wt/AUS/WAPC2016/2014/G3P[8]
84
 RVA/Human-wt/THA/SKT-281/2013/G3P[8]
97
 RVA/Human-wt/PHI/TGO12-045/2012/G1P[8]
 RVA/Human-wt/PHI/TGO12-012/2012/G1P[8]
 RVA/Human-wt/ESP/SS96217158/2015/G3P[8]
 RVA/Human-wt/HUN/ERN8187/2015/G3P[8]
97
 RVA/Human-wt/ESP/SS98242319/2015/G3P[8]
100
 RVA/Human/JPN/S13-30/2013/G3P[4]
 RVA/Human-wt/AUS/CK20048/2011/G2P[4]
100
 RVA/Human-wt/AUS/CK20055/2010/G2P[4]
 RVA/Human-wt/VNM/SP355/2013/G2P[4]
 RVA/Human-wt/VNM/SP026/2012/G1P[8]
98
Lineage IVa
 RVA/Human-wt/VNM/SP071/2012/G1P[8]
100
 RVA/Human-wt/HUN/ERN5523/2012/G3P(4)
99
 RVA/Human-wt/THA/SSKT-133/2013/G2P[4]
 RVA/Human-wt/THA/NP-M51/2013/G2P[4]
99
88
 RVA/Human-wt/THA/SKT-138/2013/G2P[4]
 RVA/Human-wt/AUS/CK20037/2008/G2P[4]
 RVA/Human-wt/THA/CU436-KK/2009/G2P[4]
73
 RVA/Human-wt/USA/LB2772/2005/G2P[4]
99
 RVA/Human-wt/VNM/SP108/2012/G2P[4]
 RVA/Human-wt/VNM/SP015/2012/G2P[4]
100
 RVA/Human-wt/VNM/NT0494/2008/G2P[4]
97
 RVA/Human-wt/ITA/PA108/2007/G2P[4]
93
100
 RVA/Human-wt/AUS/CK20030/2006/G2P[4]
99
 RVA/Human-wt/PHI/TGO12-016/2012/G1P[8]
 RVA/Human-wt/PHI/TGO12-004/2012/G1P[8]
99
 RVA/Human-wt/PHI/TGO12-003/2012/G2P[4]
76
 RVA/Human-wt/PHI/TGO12-007/2012/G2P[4]
 RVA/Human-wt/CHN/TB-Chen/1996/G2P[4]
Lineage IVnon-a
 RVA/Human-tc/JPN/KUN/1980/G2P[4]
Lineage III
 RVA/Human-tc/USA/DS-1/1976/G2P[4]
Lineage I
0.005
Supplementary Fig. 2b: VP2 gene: C2
Outgroup

## Slide 4
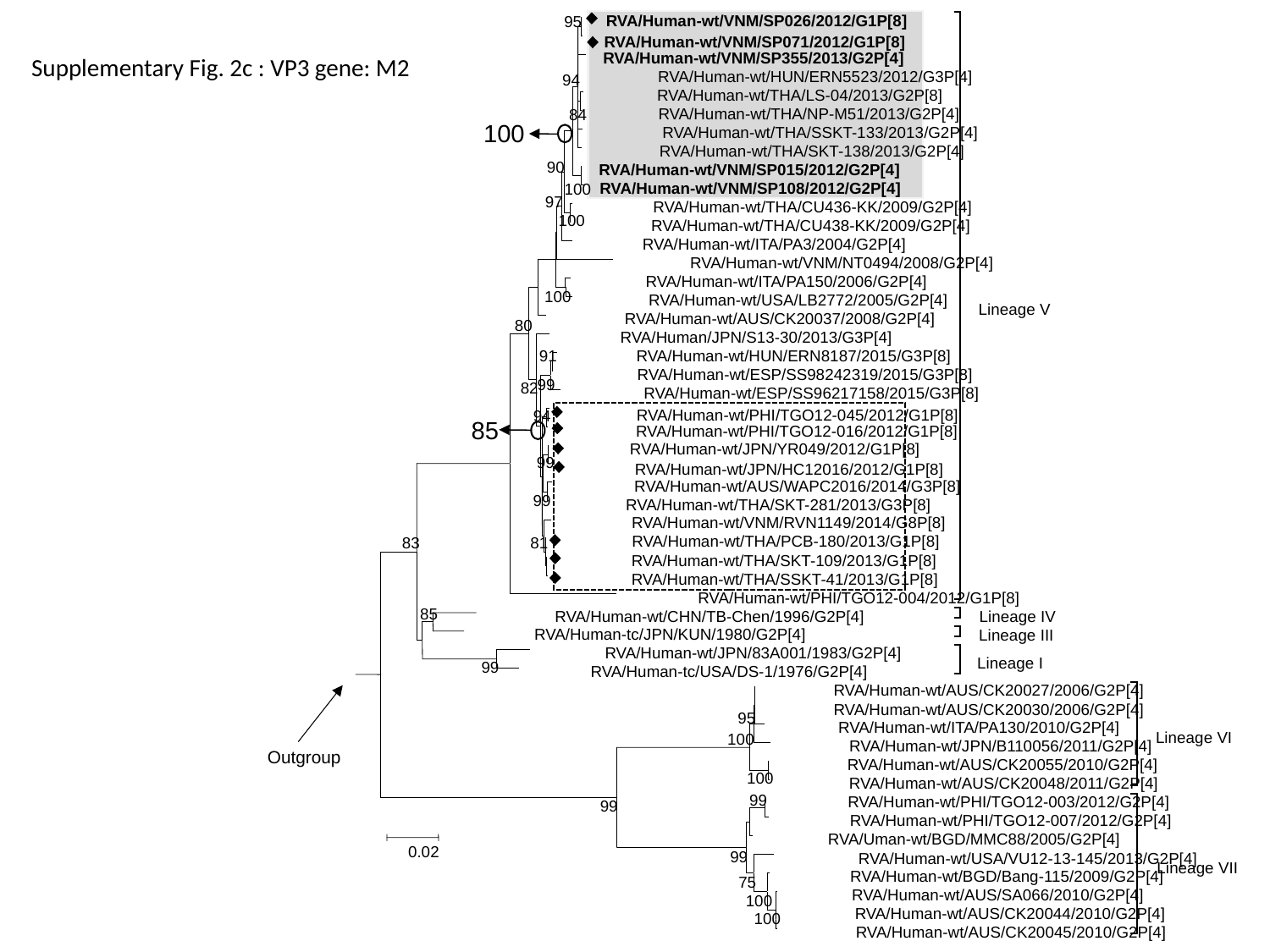

RVA/Human-wt/VNM/SP026/2012/G1P[8]
95
 RVA/Human-wt/VNM/SP071/2012/G1P[8]
 RVA/Human-wt/VNM/SP355/2013/G2P[4]
 RVA/Human-wt/HUN/ERN5523/2012/G3P[4]
94
 RVA/Human-wt/THA/LS-04/2013/G2P[8]
 RVA/Human-wt/THA/NP-M51/2013/G2P[4]
84
100
 RVA/Human-wt/THA/SSKT-133/2013/G2P[4]
 RVA/Human-wt/THA/SKT-138/2013/G2P[4]
90
 RVA/Human-wt/VNM/SP015/2012/G2P[4]
 RVA/Human-wt/VNM/SP108/2012/G2P[4]
100
97
 RVA/Human-wt/THA/CU436-KK/2009/G2P[4]
100
 RVA/Human-wt/THA/CU438-KK/2009/G2P[4]
 RVA/Human-wt/ITA/PA3/2004/G2P[4]
 RVA/Human-wt/VNM/NT0494/2008/G2P[4]
 RVA/Human-wt/ITA/PA150/2006/G2P[4]
100
 RVA/Human-wt/USA/LB2772/2005/G2P[4]
Lineage V
 RVA/Human-wt/AUS/CK20037/2008/G2P[4]
80
 RVA/Human/JPN/S13-30/2013/G3P[4]
 RVA/Human-wt/HUN/ERN8187/2015/G3P[8]
91
 RVA/Human-wt/ESP/SS98242319/2015/G3P[8]
99
82
 RVA/Human-wt/ESP/SS96217158/2015/G3P[8]
 RVA/Human-wt/PHI/TGO12-045/2012/G1P[8]
94
85
 RVA/Human-wt/PHI/TGO12-016/2012/G1P[8]
 RVA/Human-wt/JPN/YR049/2012/G1P[8]
99
 RVA/Human-wt/JPN/HC12016/2012/G1P[8]
 RVA/Human-wt/AUS/WAPC2016/2014/G3P[8]
99
 RVA/Human-wt/THA/SKT-281/2013/G3P[8]
 RVA/Human-wt/VNM/RVN1149/2014/G8P[8]
 RVA/Human-wt/THA/PCB-180/2013/G1P[8]
83
81
 RVA/Human-wt/THA/SKT-109/2013/G1P[8]
 RVA/Human-wt/THA/SSKT-41/2013/G1P[8]
 RVA/Human-wt/PHI/TGO12-004/2012/G1P[8]
85
 RVA/Human-wt/CHN/TB-Chen/1996/G2P[4]
Lineage IV
 RVA/Human-tc/JPN/KUN/1980/G2P[4]
Lineage III
 RVA/Human-wt/JPN/83A001/1983/G2P[4]
Lineage I
99
 RVA/Human-tc/USA/DS-1/1976/G2P[4]
 RVA/Human-wt/AUS/CK20027/2006/G2P[4]
 RVA/Human-wt/AUS/CK20030/2006/G2P[4]
95
 RVA/Human-wt/ITA/PA130/2010/G2P[4]
Lineage VI
100
 RVA/Human-wt/JPN/B110056/2011/G2P[4]
 RVA/Human-wt/AUS/CK20055/2010/G2P[4]
100
 RVA/Human-wt/AUS/CK20048/2011/G2P[4]
99
 RVA/Human-wt/PHI/TGO12-003/2012/G2P[4]
99
 RVA/Human-wt/PHI/TGO12-007/2012/G2P[4]
 RVA/Uman-wt/BGD/MMC88/2005/G2P[4]
0.02
99
 RVA/Human-wt/USA/VU12-13-145/2013/G2P[4]
Lineage VII
 RVA/Human-wt/BGD/Bang-115/2009/G2P[4]
75
 RVA/Human-wt/AUS/SA066/2010/G2P[4]
100
 RVA/Human-wt/AUS/CK20044/2010/G2P[4]
100
 RVA/Human-wt/AUS/CK20045/2010/G2P[4]
Supplementary Fig. 2c : VP3 gene: M2
Outgroup

## Slide 5
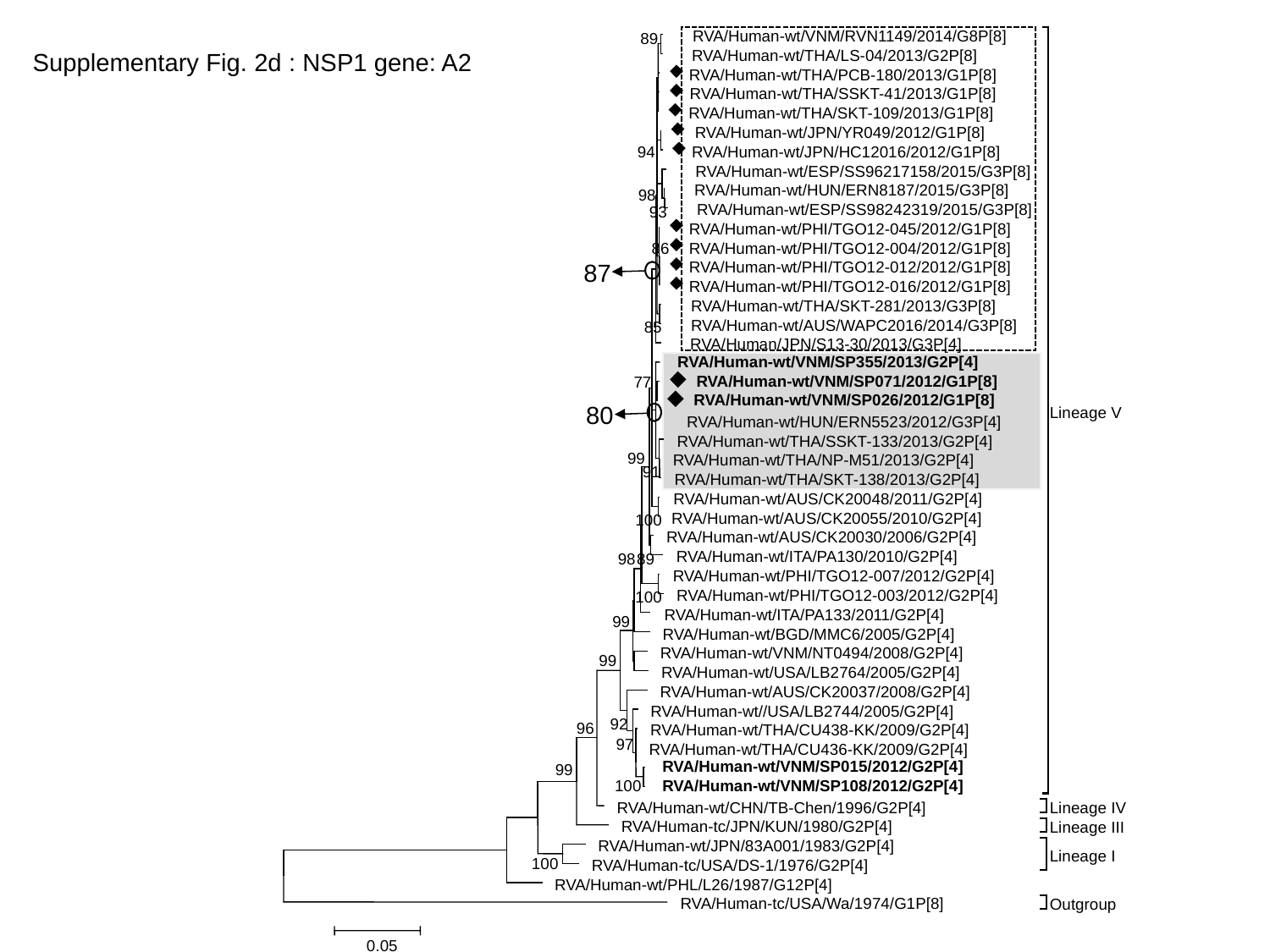

RVA/Human-wt/VNM/RVN1149/2014/G8P[8]
89
Supplementary Fig. 2d : NSP1 gene: A2
 RVA/Human-wt/THA/LS-04/2013/G2P[8]
 RVA/Human-wt/THA/PCB-180/2013/G1P[8]
 RVA/Human-wt/THA/SSKT-41/2013/G1P[8]
 RVA/Human-wt/THA/SKT-109/2013/G1P[8]
 RVA/Human-wt/JPN/YR049/2012/G1P[8]
94
 RVA/Human-wt/JPN/HC12016/2012/G1P[8]
 RVA/Human-wt/ESP/SS96217158/2015/G3P[8]
 RVA/Human-wt/HUN/ERN8187/2015/G3P[8]
98
 RVA/Human-wt/ESP/SS98242319/2015/G3P[8]
93
 RVA/Human-wt/PHI/TGO12-045/2012/G1P[8]
 RVA/Human-wt/PHI/TGO12-004/2012/G1P[8]
86
 RVA/Human-wt/PHI/TGO12-012/2012/G1P[8]
87
 RVA/Human-wt/PHI/TGO12-016/2012/G1P[8]
 RVA/Human-wt/THA/SKT-281/2013/G3P[8]
 RVA/Human-wt/AUS/WAPC2016/2014/G3P[8]
85
 RVA/Human/JPN/S13-30/2013/G3P[4]
 RVA/Human-wt/VNM/SP355/2013/G2P[4]
 RVA/Human-wt/VNM/SP071/2012/G1P[8]
77
 RVA/Human-wt/VNM/SP026/2012/G1P[8]
80
Lineage V
 RVA/Human-wt/HUN/ERN5523/2012/G3P[4]
 RVA/Human-wt/THA/SSKT-133/2013/G2P[4]
99
 RVA/Human-wt/THA/NP-M51/2013/G2P[4]
91
 RVA/Human-wt/THA/SKT-138/2013/G2P[4]
 RVA/Human-wt/AUS/CK20048/2011/G2P[4]
 RVA/Human-wt/AUS/CK20055/2010/G2P[4]
100
 RVA/Human-wt/AUS/CK20030/2006/G2P[4]
 RVA/Human-wt/ITA/PA130/2010/G2P[4]
89
98
 RVA/Human-wt/PHI/TGO12-007/2012/G2P[4]
 RVA/Human-wt/PHI/TGO12-003/2012/G2P[4]
100
 RVA/Human-wt/ITA/PA133/2011/G2P[4]
99
 RVA/Human-wt/BGD/MMC6/2005/G2P[4]
 RVA/Human-wt/VNM/NT0494/2008/G2P[4]
99
 RVA/Human-wt/USA/LB2764/2005/G2P[4]
 RVA/Human-wt/AUS/CK20037/2008/G2P[4]
 RVA/Human-wt//USA/LB2744/2005/G2P[4]
92
96
 RVA/Human-wt/THA/CU438-KK/2009/G2P[4]
97
 RVA/Human-wt/THA/CU436-KK/2009/G2P[4]
 RVA/Human-wt/VNM/SP015/2012/G2P[4]
99
100
 RVA/Human-wt/VNM/SP108/2012/G2P[4]
 RVA/Human-wt/CHN/TB-Chen/1996/G2P[4]
Lineage IV
 RVA/Human-tc/JPN/KUN/1980/G2P[4]
Lineage III
 RVA/Human-wt/JPN/83A001/1983/G2P[4]
Lineage I
100
 RVA/Human-tc/USA/DS-1/1976/G2P[4]
 RVA/Human-wt/PHL/L26/1987/G12P[4]
 RVA/Human-tc/USA/Wa/1974/G1P[8]
Outgroup
0.05

## Slide 6
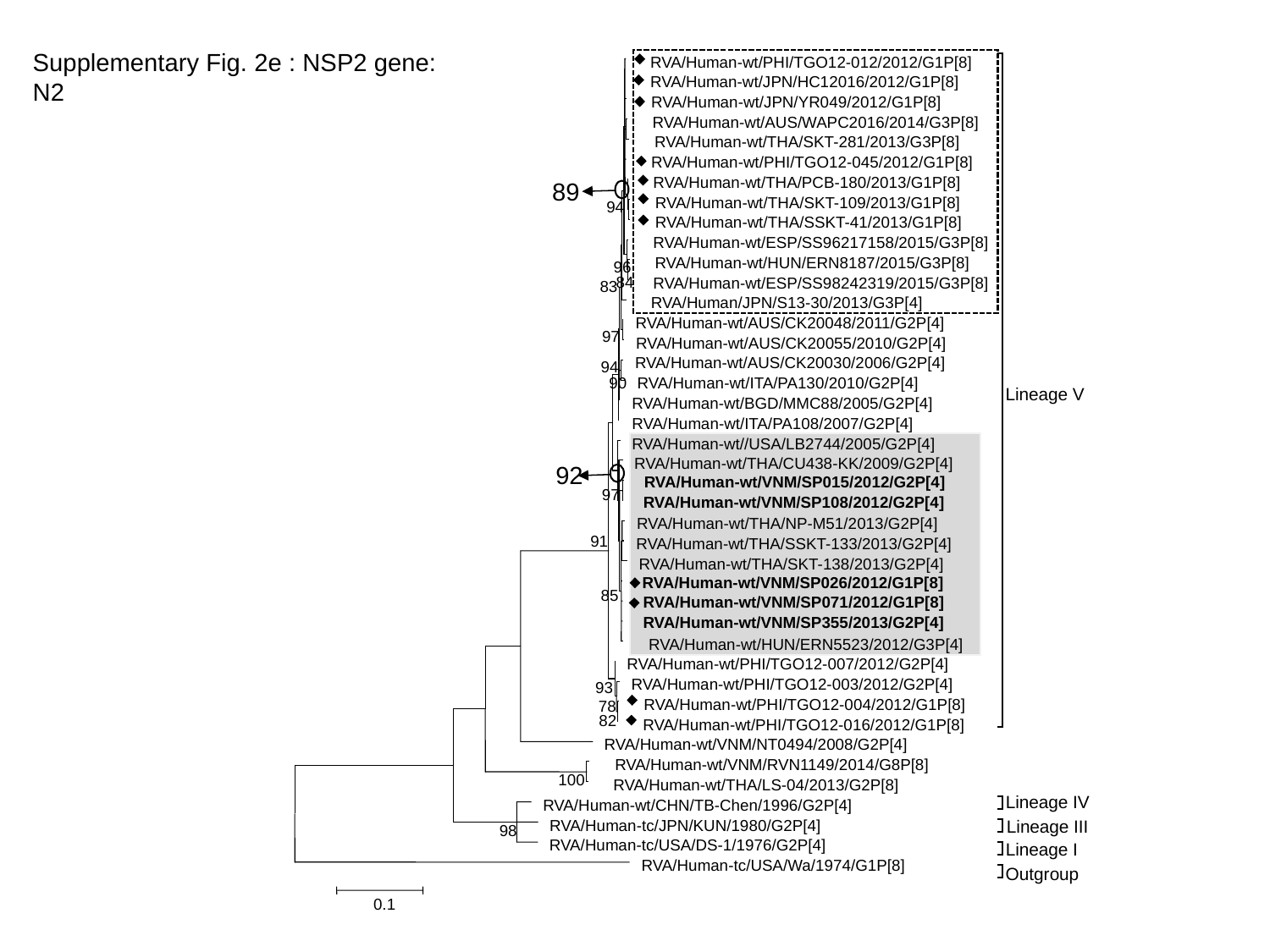

Supplementary Fig. 2e : NSP2 gene: N2
 RVA/Human-wt/PHI/TGO12-012/2012/G1P[8]
 RVA/Human-wt/JPN/HC12016/2012/G1P[8]
 RVA/Human-wt/JPN/YR049/2012/G1P[8]
 RVA/Human-wt/AUS/WAPC2016/2014/G3P[8]
 RVA/Human-wt/THA/SKT-281/2013/G3P[8]
 RVA/Human-wt/PHI/TGO12-045/2012/G1P[8]
 RVA/Human-wt/THA/PCB-180/2013/G1P[8]
89
 RVA/Human-wt/THA/SKT-109/2013/G1P[8]
94
 RVA/Human-wt/THA/SSKT-41/2013/G1P[8]
 RVA/Human-wt/ESP/SS96217158/2015/G3P[8]
 RVA/Human-wt/HUN/ERN8187/2015/G3P[8]
96
84
 RVA/Human-wt/ESP/SS98242319/2015/G3P[8]
83
 RVA/Human/JPN/S13-30/2013/G3P[4]
 RVA/Human-wt/AUS/CK20048/2011/G2P[4]
97
 RVA/Human-wt/AUS/CK20055/2010/G2P[4]
 RVA/Human-wt/AUS/CK20030/2006/G2P[4]
94
90
 RVA/Human-wt/ITA/PA130/2010/G2P[4]
Lineage V
 RVA/Human-wt/BGD/MMC88/2005/G2P[4]
 RVA/Human-wt/ITA/PA108/2007/G2P[4]
 RVA/Human-wt//USA/LB2744/2005/G2P[4]
 RVA/Human-wt/THA/CU438-KK/2009/G2P[4]
92
 RVA/Human-wt/VNM/SP015/2012/G2P[4]
97
 RVA/Human-wt/VNM/SP108/2012/G2P[4]
 RVA/Human-wt/THA/NP-M51/2013/G2P[4]
91
 RVA/Human-wt/THA/SSKT-133/2013/G2P[4]
 RVA/Human-wt/THA/SKT-138/2013/G2P[4]
 RVA/Human-wt/VNM/SP026/2012/G1P[8]
85
 RVA/Human-wt/VNM/SP071/2012/G1P[8]
 RVA/Human-wt/VNM/SP355/2013/G2P[4]
 RVA/Human-wt/HUN/ERN5523/2012/G3P[4]
 RVA/Human-wt/PHI/TGO12-007/2012/G2P[4]
 RVA/Human-wt/PHI/TGO12-003/2012/G2P[4]
93
 RVA/Human-wt/PHI/TGO12-004/2012/G1P[8]
78
82
 RVA/Human-wt/PHI/TGO12-016/2012/G1P[8]
 RVA/Human-wt/VNM/NT0494/2008/G2P[4]
 RVA/Human-wt/VNM/RVN1149/2014/G8P[8]
100
 RVA/Human-wt/THA/LS-04/2013/G2P[8]
Lineage IV
Lineage III
Lineage I
Outgroup
 RVA/Human-wt/CHN/TB-Chen/1996/G2P[4]
 RVA/Human-tc/JPN/KUN/1980/G2P[4]
98
 RVA/Human-tc/USA/DS-1/1976/G2P[4]
 RVA/Human-tc/USA/Wa/1974/G1P[8]
0.1

## Slide 7
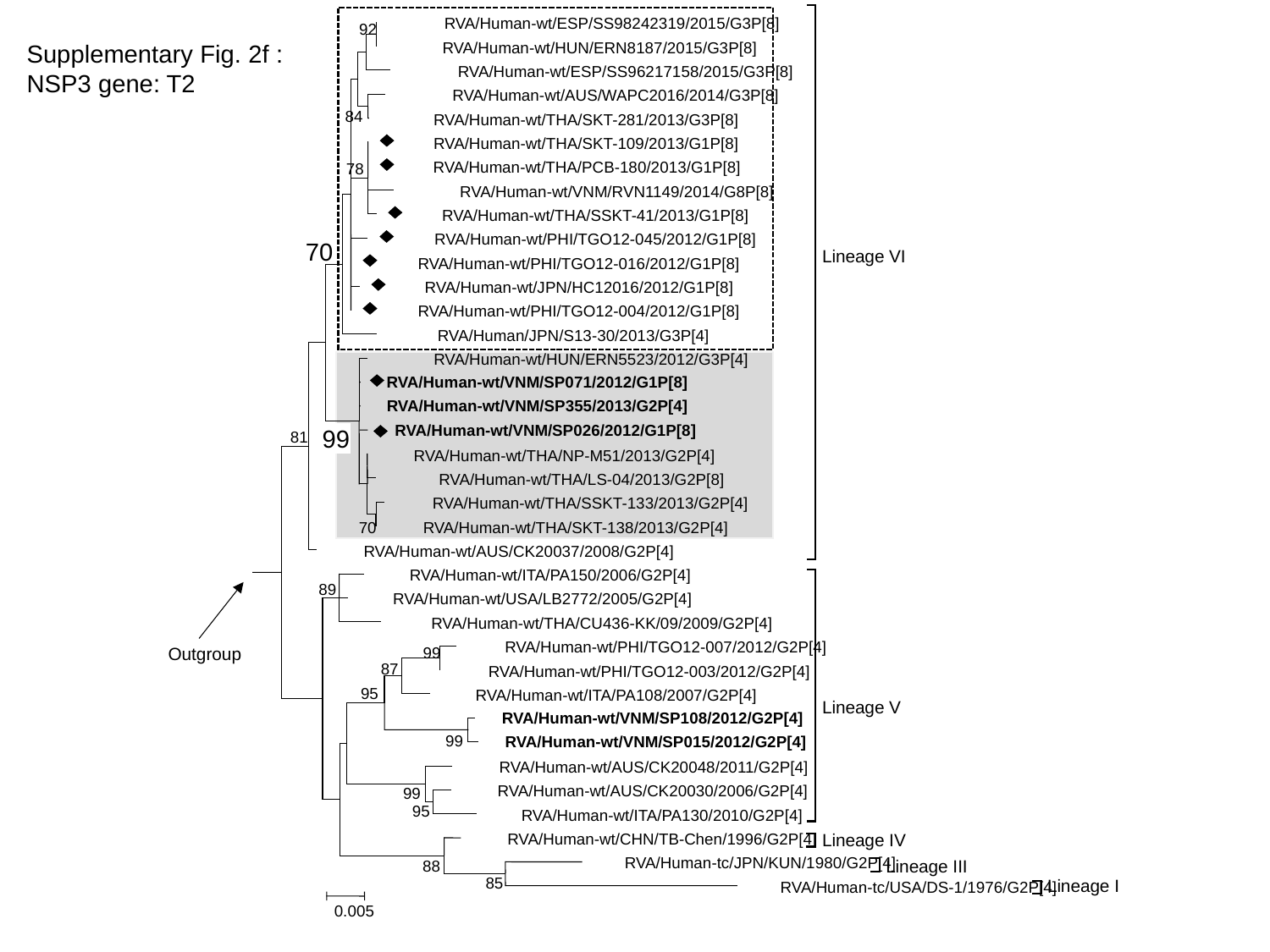

RVA/Human-wt/ESP/SS98242319/2015/G3P[8]
92
 RVA/Human-wt/HUN/ERN8187/2015/G3P[8]
 RVA/Human-wt/ESP/SS96217158/2015/G3P[8]
 RVA/Human-wt/AUS/WAPC2016/2014/G3P[8]
84
 RVA/Human-wt/THA/SKT-281/2013/G3P[8]
 RVA/Human-wt/THA/SKT-109/2013/G1P[8]
 RVA/Human-wt/THA/PCB-180/2013/G1P[8]
78
 RVA/Human-wt/VNM/RVN1149/2014/G8P[8]
 RVA/Human-wt/THA/SSKT-41/2013/G1P[8]
 RVA/Human-wt/PHI/TGO12-045/2012/G1P[8]
70
 RVA/Human-wt/PHI/TGO12-016/2012/G1P[8]
 RVA/Human-wt/JPN/HC12016/2012/G1P[8]
 RVA/Human-wt/PHI/TGO12-004/2012/G1P[8]
 RVA/Human/JPN/S13-30/2013/G3P[4]
 RVA/Human-wt/HUN/ERN5523/2012/G3P[4]
 RVA/Human-wt/VNM/SP071/2012/G1P[8]
 RVA/Human-wt/VNM/SP355/2013/G2P[4]
 RVA/Human-wt/VNM/SP026/2012/G1P[8]
99
81
 RVA/Human-wt/THA/NP-M51/2013/G2P[4]
 RVA/Human-wt/THA/LS-04/2013/G2P[8]
 RVA/Human-wt/THA/SSKT-133/2013/G2P[4]
 RVA/Human-wt/THA/SKT-138/2013/G2P[4]
70
 RVA/Human-wt/AUS/CK20037/2008/G2P[4]
 RVA/Human-wt/ITA/PA150/2006/G2P[4]
89
 RVA/Human-wt/USA/LB2772/2005/G2P[4]
 RVA/Human-wt/THA/CU436-KK/09/2009/G2P[4]
 RVA/Human-wt/PHI/TGO12-007/2012/G2P[4]
99
87
 RVA/Human-wt/PHI/TGO12-003/2012/G2P[4]
95
 RVA/Human-wt/ITA/PA108/2007/G2P[4]
 RVA/Human-wt/VNM/SP108/2012/G2P[4]
99
 RVA/Human-wt/VNM/SP015/2012/G2P[4]
 RVA/Human-wt/AUS/CK20048/2011/G2P[4]
 RVA/Human-wt/AUS/CK20030/2006/G2P[4]
99
95
 RVA/Human-wt/ITA/PA130/2010/G2P[4]
 RVA/Human-wt/CHN/TB-Chen/1996/G2P[4]
 RVA/Human-tc/JPN/KUN/1980/G2P[4]
88
85
 RVA/Human-tc/USA/DS-1/1976/G2P[4]
0.005
Supplementary Fig. 2f : NSP3 gene: T2
Lineage VI
Outgroup
Lineage V
Lineage IV
Lineage III
Lineage I

## Slide 8
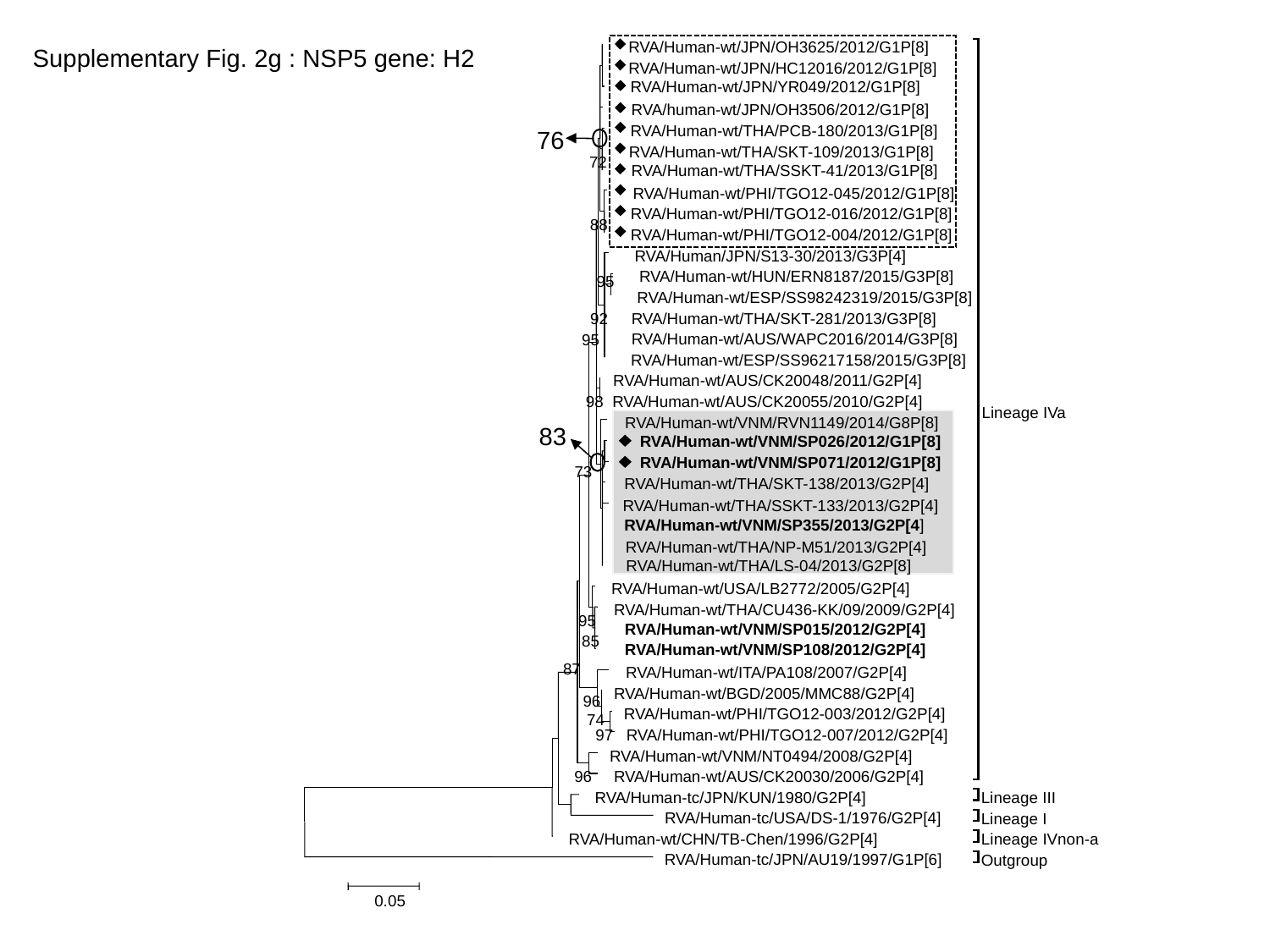

Supplementary Fig. 2g : NSP5 gene: H2
 RVA/Human-wt/JPN/OH3625/2012/G1P[8]
 RVA/Human-wt/JPN/HC12016/2012/G1P[8]
 RVA/Human-wt/JPN/YR049/2012/G1P[8]
 RVA/human-wt/JPN/OH3506/2012/G1P[8]
 RVA/Human-wt/THA/PCB-180/2013/G1P[8]
76
 RVA/Human-wt/THA/SKT-109/2013/G1P[8]
72
 RVA/Human-wt/THA/SSKT-41/2013/G1P[8]
 RVA/Human-wt/PHI/TGO12-045/2012/G1P[8]
 RVA/Human-wt/PHI/TGO12-016/2012/G1P[8]
88
 RVA/Human-wt/PHI/TGO12-004/2012/G1P[8]
 RVA/Human/JPN/S13-30/2013/G3P[4]
 RVA/Human-wt/HUN/ERN8187/2015/G3P[8]
95
 RVA/Human-wt/ESP/SS98242319/2015/G3P[8]
 RVA/Human-wt/THA/SKT-281/2013/G3P[8]
92
 RVA/Human-wt/AUS/WAPC2016/2014/G3P[8]
95
 RVA/Human-wt/ESP/SS96217158/2015/G3P[8]
 RVA/Human-wt/AUS/CK20048/2011/G2P[4]
 RVA/Human-wt/AUS/CK20055/2010/G2P[4]
98
Lineage IVa
 RVA/Human-wt/VNM/RVN1149/2014/G8P[8]
83
 RVA/Human-wt/VNM/SP026/2012/G1P[8]
 RVA/Human-wt/VNM/SP071/2012/G1P[8]
73
 RVA/Human-wt/THA/SKT-138/2013/G2P[4]
 RVA/Human-wt/THA/SSKT-133/2013/G2P[4]
 RVA/Human-wt/VNM/SP355/2013/G2P[4]
 RVA/Human-wt/THA/NP-M51/2013/G2P[4]
 RVA/Human-wt/THA/LS-04/2013/G2P[8]
 RVA/Human-wt/USA/LB2772/2005/G2P[4]
 RVA/Human-wt/THA/CU436-KK/09/2009/G2P[4]
95
 RVA/Human-wt/VNM/SP015/2012/G2P[4]
85
 RVA/Human-wt/VNM/SP108/2012/G2P[4]
87
 RVA/Human-wt/ITA/PA108/2007/G2P[4]
 RVA/Human-wt/BGD/2005/MMC88/G2P[4]
96
 RVA/Human-wt/PHI/TGO12-003/2012/G2P[4]
74
 RVA/Human-wt/PHI/TGO12-007/2012/G2P[4]
97
 RVA/Human-wt/VNM/NT0494/2008/G2P[4]
 RVA/Human-wt/AUS/CK20030/2006/G2P[4]
96
 RVA/Human-tc/JPN/KUN/1980/G2P[4]
Lineage III
 RVA/Human-tc/USA/DS-1/1976/G2P[4]
Lineage I
 RVA/Human-wt/CHN/TB-Chen/1996/G2P[4]
Lineage IVnon-a
 RVA/Human-tc/JPN/AU19/1997/G1P[6]
Outgroup
0.05
